# Variability in the functional composition of coral reef fish communities on submerged and emergent reefs in the central Great Barrier Reef, Australia

**DOI:** 10.1371/journal.pone.0216785

**Published:** 2019-05-17

**Authors:** Amanda M. Cooper, Chancey MacDonald, T. Edward Roberts, Tom C. L. Bridge

**Affiliations:** 1 Marine Biology and Aquaculture Science, College of Science and Engineering, James Cook University, Townsville, Queensland, Australia; 2 Australian Research Council Centre of Excellence in Coral Reef Studies, James Cook University, Townsville, Queensland, Australia; 3 Queensland Museum Network, Townsville, Queensland, Australia; Swansea University, UNITED KINGDOM

## Abstract

On coral reefs, depth and gradients related to depth (e.g. light and wave exposure) influence the composition of fish communities. However, most studies focus only on emergent reefs that break the sea surface in shallow waters (<10 m). On the Great Barrier Reef (GBR), submerged reefs (reefs that do not break the sea surface) occupy an area equivalent to all emergent reefs. However, submerged reefs have received comparatively little research attention, and fish communities associated with submerged reefs remain poorly quantified. Here, we quantify fish assemblages at each of three depths (10, 20 and 30 m) on eight submerged reefs (four mid-shelf and four outer-shelf) and two nearby emergent reefs in the central GBR where reef habitat extends from 0-~25 m depth. We examine how total fish abundance, the abundance of 13 functional groups, and the functional composition of fish communities varies among depths, reef types (submerged versus emergent reefs), and shelf position (mid-shelf versus outer-shelf). Overall fish abundance decreased sevenfold with depth, but declined less steeply (twofold) on outer-shelf submerged reefs than on both mid-shelf submerged reefs and emergent reefs. The functional composition of the fish assemblage also varied significantly among depths and reef types. Turnover in the functional composition of the fish community was also steeper on the mid-shelf, suggesting that shallow-affiliated groups extend further in deeper water on the outer-shelf. Ten of the 13 functional groups were more strongly associated with the shallowest depths (the upper reef slope of emergent reefs or the ‘crests’ of submerged reefs), two groups (soft coral/sponge feeders and mesopredators) were more abundant at the deepest sites. Our results confirm that submerged reefs in the central GBR support a wide range of coral reef fishes, and are an important component of the GBR ecosystem.

## Introduction

Ecological gradients such as latitude, depth, altitude and exposure exert a strong influence on the distribution and abundance of species [1]. In marine ecosystems, depth and gradients related to depth, such as light and temperature, influence the abundance and spatial distribution of fish assemblages [2–5]. Depth has been shown to influence reef fish distributions at all life history stages, from larval [6], to settlement and recruitment [7, 8], and post-settlement phases [9, 10]. However, these responses vary between families and species [8, 11]. Monitoring functional groups, species that perform similar roles within an ecosystem [12], regardless of taxonomic affinity [13, 14], can enable the detection of changes within a reef ecosystem through the understanding of ecological processes and gradients that may be overlooked using traditional nomenclatorial approaches based on taxonomic identities [15, 16].

Biotic and abiotic habitat characteristics, which may be correlated with depth, are also an important factor shaping the composition of functional groups of reef fishes [17–19]. For example, diminishing light levels with increasing depth results in decreased algal growth [20], altered foraging behaviour of mobile species [21], and modified habitat complexity through changing coral assemblages [5, 8]. Differences in structural complexity [22, 23] and nutrition [24] of deep-water corals may also affect their suitability as habitats for different functional groups. The relationship between depth and other key determinants of fish distributions makes identifying underlying causes of depth-diversity gradients in reef fish assemblages difficult. This problem is exacerbated by the inherent difficulties in accessing and collecting data from deeper reefs, which has led to the vast majority of studies on reef fish being conducted in shallow waters. Consequently, how and why coral reef fish communities and functional assemblages change along depth gradients remains poorly understood, despite depth range being a key determinant of extinction risk for coral reef fishes [25].

On the Great Barrier Reef (GBR), key environmental factors that influence coral reef communities and ecosystem functions vary substantially across the continental shelf; reefs in close proximity to the coast are heavily influenced by terrestrial runoff and sedimentation, while those offshore occur in the clear, oligotrophic waters of the Coral Sea [26–28]. Cross-shelf gradients in the physical environment result in concomitant changes in the composition and abundance of reef-associated benthos and associated fish communities [29–32]. Changes in the functional composition of reef fish assemblages along cross-shelf and depth gradients strongly influence key ecological processes, such as herbivory, assisting in reef resilience by preventing coral-algal phase shifts [33–35].

To date, the vast majority of ecological research on the GBR has occurred on shallow emergent reefs. However, there is increasing recognition that the GBR also supports vast quantities of submerged reefs that do not break sea level [36–38]. Submerged reefs are defined by the International Hydrographic Organization as an “isolated elevation of the seafloor, over which the depth of water is relatively shallow but sufficient for navigation” [39]. In the central GBR, many submerged reefs rise to within 10–15 m of the sea surface, enabling them to support profuse growth of stony corals with similar composition to nearby shallow-water reefs. Roberts et al. [38] examined benthic communities on submerged and nearby emergent reefs in the central GBR. As expected, benthic community composition changed considerably with depth and across the shelf [38]. In addition, similar coral communities generally occurred deeper on submerged reefs than on nearby emergent reefs, a pattern attributed to differences in hydrodynamics between reef morphologies [38]. Given the important influence of depth and benthic composition on reef-associated fishes, similar changes could be expected in fish assemblages; however, the abundance and composition of fish communities associated with submerged reefs on the GBR is currently not quantified. Here, we examine: 1) how total fish abundance, the abundance of each of 13 functional groups, and the functional composition of fish communities varies both with depth and between reef morphologies (submerged versus emergent reefs) in the central GBR; and 2) the extent to which these patterns were attributable to changes in shelf position (mid-shelf versus outer-shelf), depth and benthic composition.

## Materials and methods

### Ethics statement

This research project involved only visual censuses and no fauna or flora were collected or manipulated during this study. The study was therefore classified as ‘limited impact research’, as defined by the Great Barrier Reef Marine Park Authority (GBRMPA). Since all researchers were associated with James Cook University, a GBRMPA accredited research institution, no permit was required to conduct this research project. For further information see http://www.gbrmpa.gov.au/zoning-permits-and-plans/permits/advice-on-research-permits/accredited-educational-and-research-institutions

### Study site

**Fig 1:**
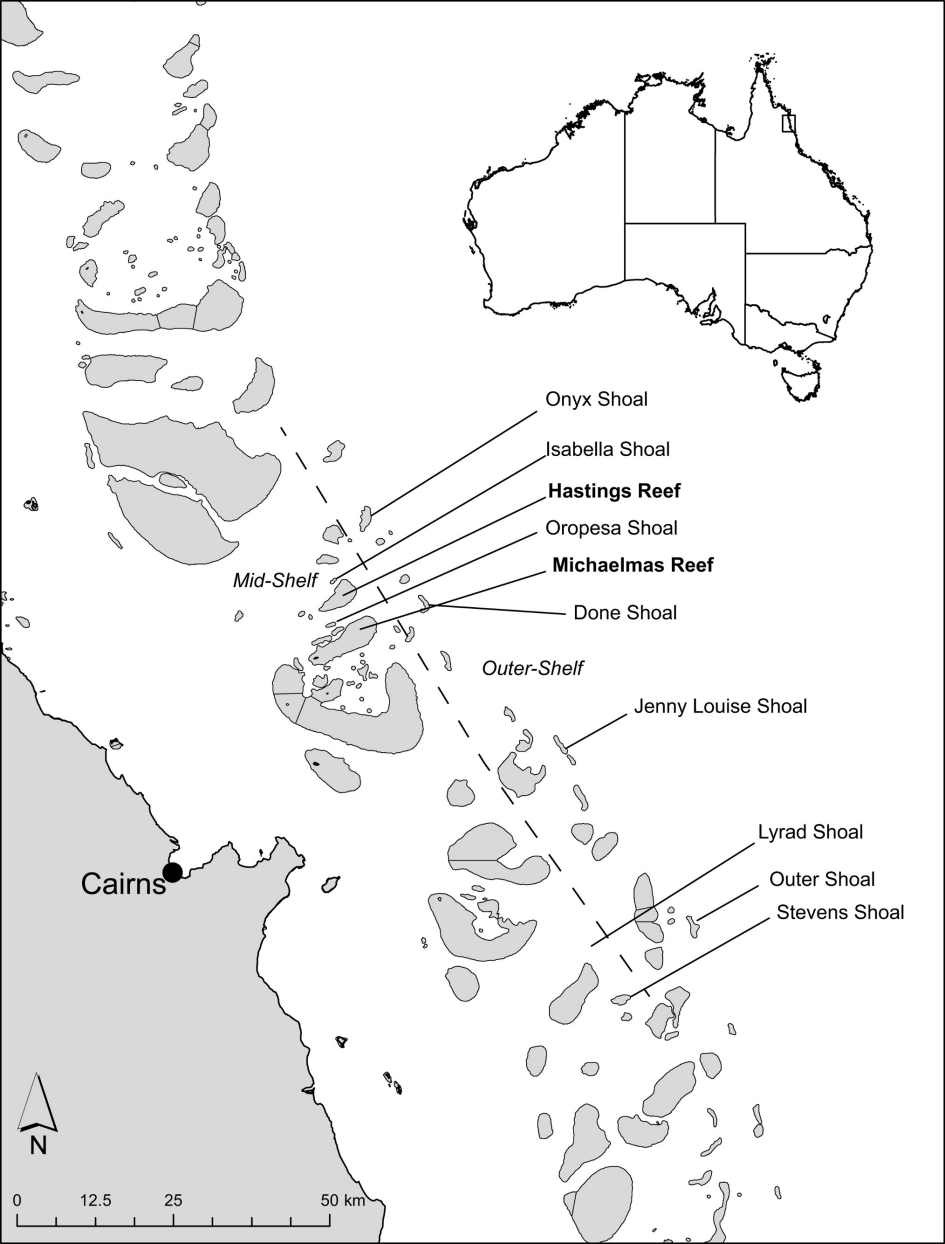
Map showing the location of study sites in the central Great Barrier Reef. Dashed line shows the boundary between mid-shelf and outer-shelf reefs. Emergent reef names are highlighted in bold typeface.

We surveyed the abundance of fishes in each of 13 functional groups: corallivores, soft coral/sponge feeders, benthic carnivores, detritivores, territorial farmers, site-attached planktivores, roving planktivores, omnivores, algal croppers, algal scrapers, excavators, mesopredators, and apex predators, based on categories used by Allen et al. [40], Cole et al. [41], and Williamson et al. [42] (S1 Table). Data were collected from eight submerged reefs in the Cairns sector of the central GBR: four on the mid-shelf (MSub) (Isabella Shoal, Lyrad Shoal, Oropesa Shoal and Stevens Shoal), four on the outer-shelf (OSub) (Done Shoal, Jenny Louise Shoal, Onyx Shoal and Outer Shoal), and two nearby mid-shelf emergent reefs (EM) (Hasting Reef and Michaelmas Reef) (Fig 1). Submerged reefs in the region occur within the GBR lagoon and along the shelf-edge, seaward of the outermost emergent reefs [37]. The region is adjacent to the Wet Tropics World Heritage Area, and the influence of several large river systems results in a strong cross-shelf turbidity gradient [28]. Submerged reefs, which were at least 10 m deep at their shallowest point, were identified using the high-resolution bathymetry model for the GBR, ‘GBR100’ [43], in combination with nautical charts. To compare patterns between submerged and emergent reefs, we surveyed two nearby emergent reefs (EM) (Hastings Reef and Michaelmas Reef), that occurred within the Cairns region, that had been monitored regularly by the Australian Institute of Marine Science (AIMS) Long-term Monitoring Program (LTMP) [44].

### Data collection

Scuba divers recorded 30 m x 4 m high definition video belt transects in February and March of 2013. On submerged reefs, transects were conducted at 10, 20 and 30 m depth (+/- 2 m). Emergent reefs, by definition, extend into shallower depths than submerged reefs, therefore, an additional transect was conducted at 6 m on emergent reefs (S2 Table). The 6 m transects on emergent reefs were conducted to allow comparison with AIMS LTMP sites, and to examine whether particular fish assemblages occurred at greater depths on submerged reefs than emergent reefs [5, 38]. Lower reef slopes on emergent reefs also merged into sand at shallower depths, precluding transects at 30 m on emergent reefs.

Fish recorded in the video transects were identified based on Allen et al. [40]. The transects were filmed with the camera facing forward to capture fish communities as the diver swam toward them. Fish in the video transects were recorded based on a standardized lower half of the computer screen, and placed into functional groups defined in Cole et al. [41], and Williamson et al. [42] (S1 Table). Recording individuals observed in the lower half of the computer screen ensured that individuals were positively identified regardless of water visibility and not counted more than once as the diver swam forward along the transect. Cryptic species (e.g. family Gobiidae and others) were not recorded due to the potential for unreliable estimates of abundance in video transects. Benthic data were recorded for each 30 m transect following Roberts et al. [38] and were grouped into morphological categories considered potentially important for influencing the composition of fish assemblages, *sensu* MacDonald et al. [5]. For example, aspects such as habitat complexity which has a strong role for sheltering fishes (e.g. Luckhurst and Luckhurst [45], Coker et al. [46], Nash et al. [22], Noonan et al. [47]) and key food items of sessile benthic feeding groups (e.g. soft corals, sponges, and turf algae) were taken into account when establishing functional/morphological substrate groupings [5, 38]. The ten benthic groups were massive coral, encrusting coral, laminar coral, complex coral, turf algae, crustose calcareous algae, soft corals and sponges, coral rubble, sand and silt, and reef matrix. Complex corals were defined as those considered to be the most suitable complex habitat for the sheltering of small reef fishes. This included all branching, corymbose, hispidose, digitate, foliose and tabulate forms, but not laminar, massive, sub-massive or encrusting corals.

### Data analysis

Both mean total fish abundance and the mean abundance of each functional group were compared among depths (6, 10, 20 and 30 m) and reef types (submerged or emergent) using analysis of variance (ANOVAs) of linear models (lm) on log-transformed data in R 3.2.1 [48]. Akaike information criterion (AIC) was used to compare these models with those that accounted for spatial structure among reefs (random factor) using the maximum likelihood (ML) method of lmer in the R package ‘lme4’ [49]. Pairwise comparisons of levels within significant factors of best-fit models were performed using Tukey’s post-hoc analyses in the R package ‘lsmeans’ [50]. To test if adding variables of benthic composition improved the predictive power of models after accounting for the variance explained by depth and reef type, total fish abundance and the abundance of each functional group were first regressed against the cover of four functionally important benthic groups, complex coral, hard coral, soft coral and sponges, and turf algae individually using generalized linear mixed-effects models (GLMM) in R [49]. One benthic component (complex coral cover) was significantly correlated with fish abundance and was subsequently included as an additional term to depth x reef type models. AIC scores were used to assess if the addition of complex coral improved model fit. For all models, assumptions of normality and homogeneity of variance were confirmed using residual plots. Two groups (apex predators and excavators) were not sufficiently abundant to confidently analyze changes among sites and depths.

Changes in the functional composition of the fish community as a whole were examined using distance-based multivariate techniques in PERMANOVA+ for PRIMER v6 [51]. All analyses were conducted using a log-transformed Bray-Curtis dissimilarity matrix of fish abundances. Variability in the functional composition of the fish assemblage at each reef/depth combination were quantified using Permutational Multivariate Analysis of Variance (PERMANOVA) [52]. Homogeneity of multivariate variance in the composition of the functional group assemblages among depths and reefs types was quantified using Permutational Analysis of Multivariate Dispersions (PERMDISP). Relationships among depths and reef types were visualized using Principal Coordinates Analysis (PCO), with vectors indicating the influence of functional groups with a Spearman Rank Correlation ≥ 0.3. BEST analysis (also in PRIMER) [53] was used to identify which combination of five environmental variables (depth, reef type, complex coral cover, soft coral/sponge cover, hard coral cover, and turf algae cover) best explained variability in the fish community among depths and reef types.

## Results

### Fish abundance and functional composition

**Fig 2:**
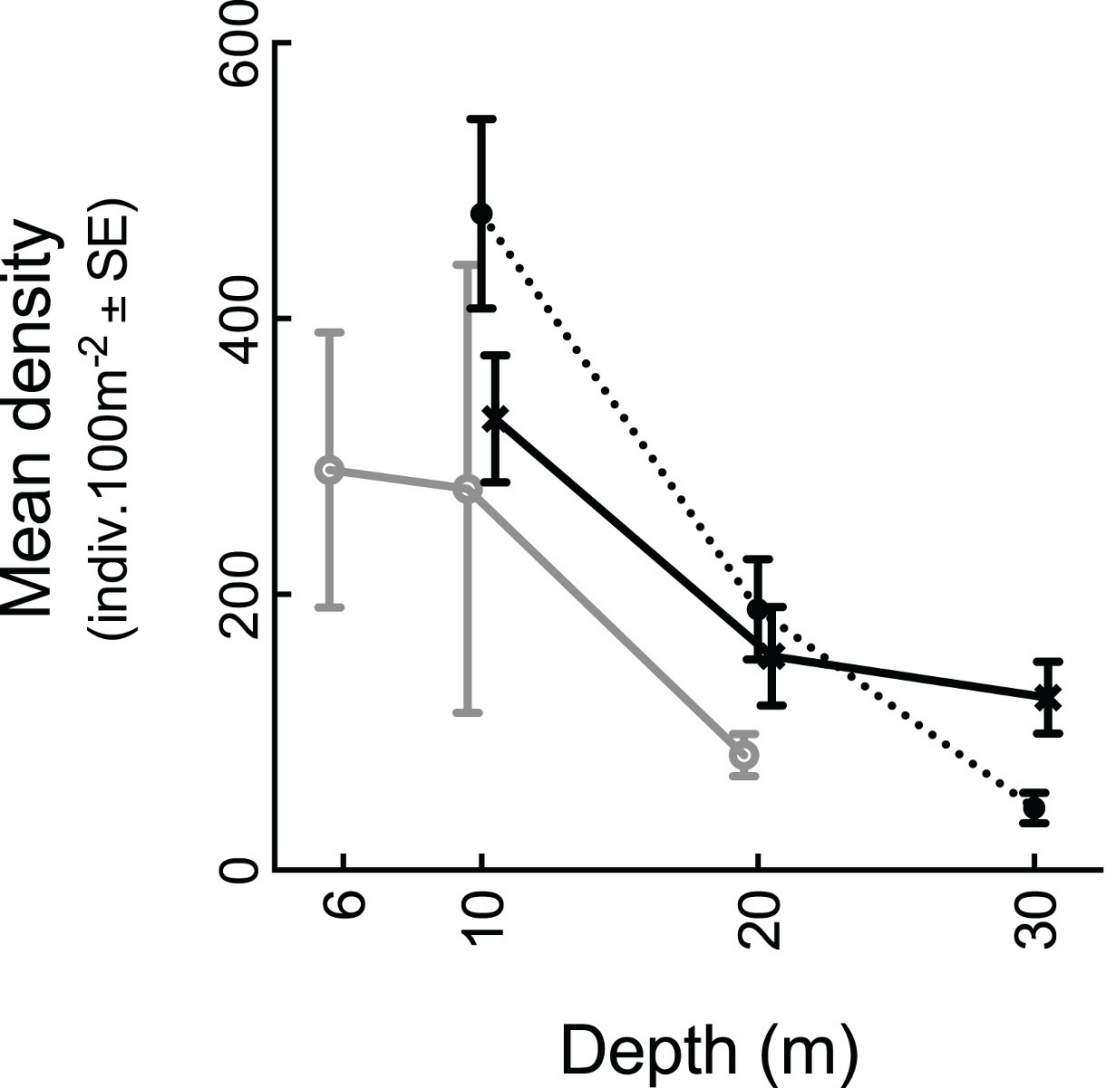
Mean total fish abundance among reef types with depths pooled. Grey line = emergent reefs, dotted black line = mid-shelf submerged reefs, solid black line = outer-shelf submerged reefs.

Mean total fish abundance varied significantly among depths (*p* = 0.001), but not reef type (Table 1A). However, 44% of variation in density distributions was explained by an interaction between depth and reef type (Table 1A). Therefore, whilst total fish abundance generally declined with depth (Fig 2), mean abundance was stable between 20 m and 30 m on outer-shelf submerged reefs (Tukey’s *p* > 0.05). Model fits of total fish abundance were not improved by accounting for spatial structure among reefs (Table 1A), but 10 of the functional groups were found to improve with the inclusion of spatial structure among reefs as a random factor (Table 1B).

**Table 1:** Summary statistics for variation in the distribution and functional assemblage of reef fishes on submerged and emergent reefs in the central section of the Great Barrier Reef.

a														
	Depth			Reef Type			Depth x Reef Type			Sig. Model	+ Complex Coral	+ Spatial Structure (Reef)	Cor. Complex Coral	
	*p*	*R*^*2*^	*Pairwise*	*p*	*R*^*2*^	*Pairwise*	*p*	*R*^*2*^	*Pair-wise*	*AIC*	*AIC*	*ΔAIC*	*p*	*R*^*2*^
Functional Assemblage	***	-	-	***	-	-	**		*T2*	-	-	-	-	-
Total Abundance	***	-	-	NS	-	-	**	**0.44**	*TS2*	-33.1	**-46.6**	0.05	***	0.21
b														
Functional Group		Model (GLMM)						AICc		ΔAICc		*wi*		
Corallivore		depth+hcc+soft.sponge+turf						**4.543**		0.98		0.091		
Soft Corallivore		depth+reeftype+soft.sponge						**35.609**		0		0.117		
Benthic Carnivore		depth+reeftype+complex.coral+hcc						**76.256**		0.067		0.246		
Detritivore		depth+reeftype						**48.272**		1.905		0.129		
Site Attached		depth+reeftype+complex.coral+soft.sponge						**129.503**		0		0.135		
Roving Planktivore		complex.coral+hcc						**247.863**		1.294		0.081		
Omnivore		depth+complex.coral+soft.sponge+turf						**96.158**		1.643		0.078		
Algal Cropper		depth+reeftype+hcc						**100.443**		0.965		0.085		
Algal Scraper		depth+reeftype+soft.sponge						**58.836**		1.56		0.113		
Excavator		depth+complex.coral+hcc+turf						-32.499		0.098		0.198		
Mesopredator		depth+turf						**59.562**		0.797		0.08		

a. Note. * indicates significant differences; * = 0.05, ** = 0.01, *** = 0.001. Bold text indicates the best explanatory variables in modeling each fish group. Bold AIC scores show models that improved with the inclusion of complex coral cover and/or the inclusion of spatial structure among reefs as a random factor. *T2* = Table 2, *TS2* = S2 Table. b. *Note*. Depth = sampling depth, hcc = hard coral cover, soft.sponge = soft sponge cover, turf = turf algal cover, reeftype = submerged reef or emergent reef, complex.coral = complex coral cover. Table describes Akaike Information Criterion (AICc), change in AIC (ΔAICc) relative to the most parsimonious model, and the model weight (*wi*). Bold AIC scores show models that improved with the inclusion of spatial structure among reefs as a random factor.

**Fig 3:**
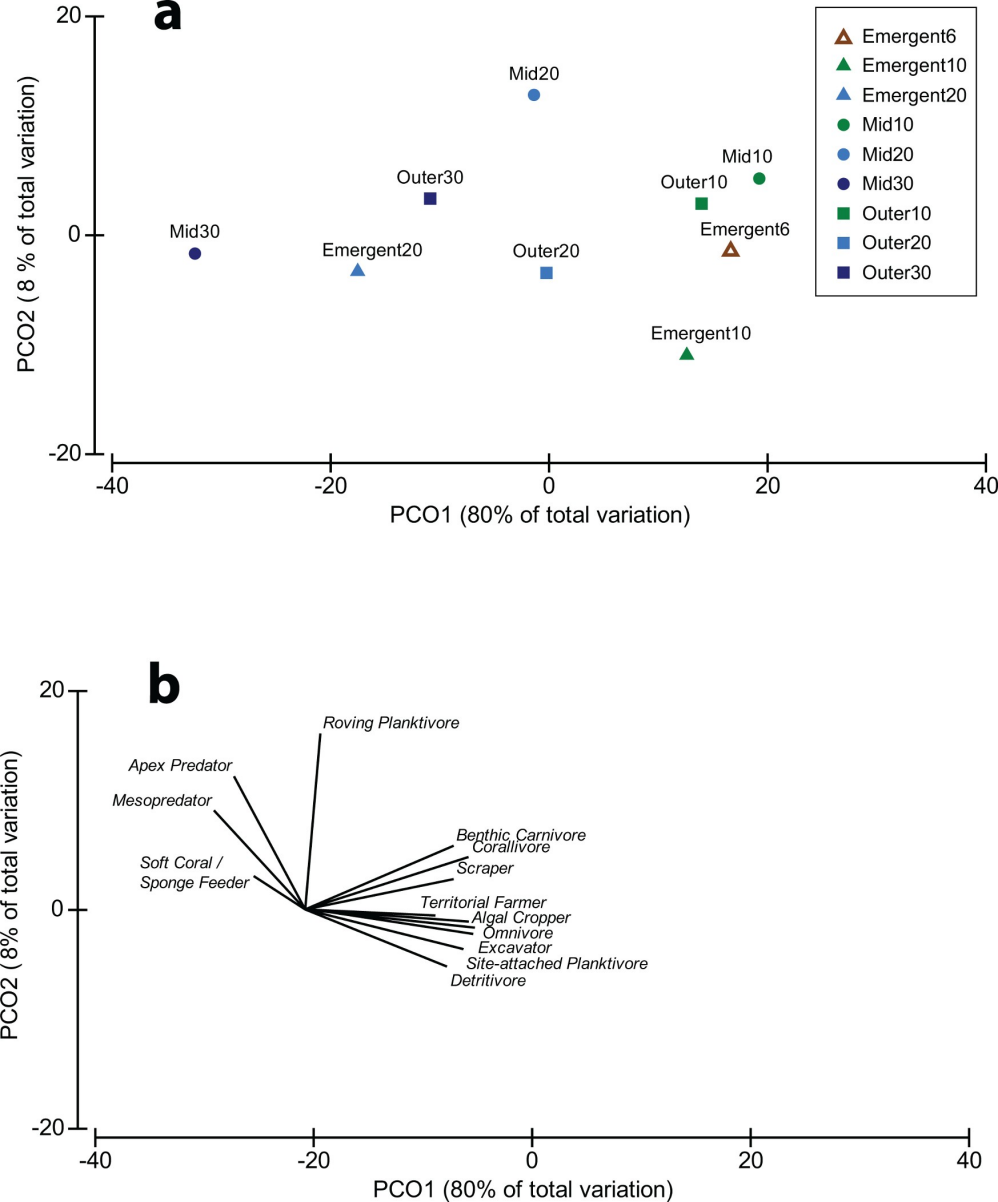
Principal coordinates plot of changes in the composition of functional assemblages of reef fishes among depths and reef types. Shapes indicate reef types (triangles = emergent reef, circles = mid-shelf submerged reef and squares = outer-shelf submerged reef), while colours indicate different depths (dark blue = 30 m, light blue = 20 m, green = 10 m and brown = 6 m).

**Fig 4:**
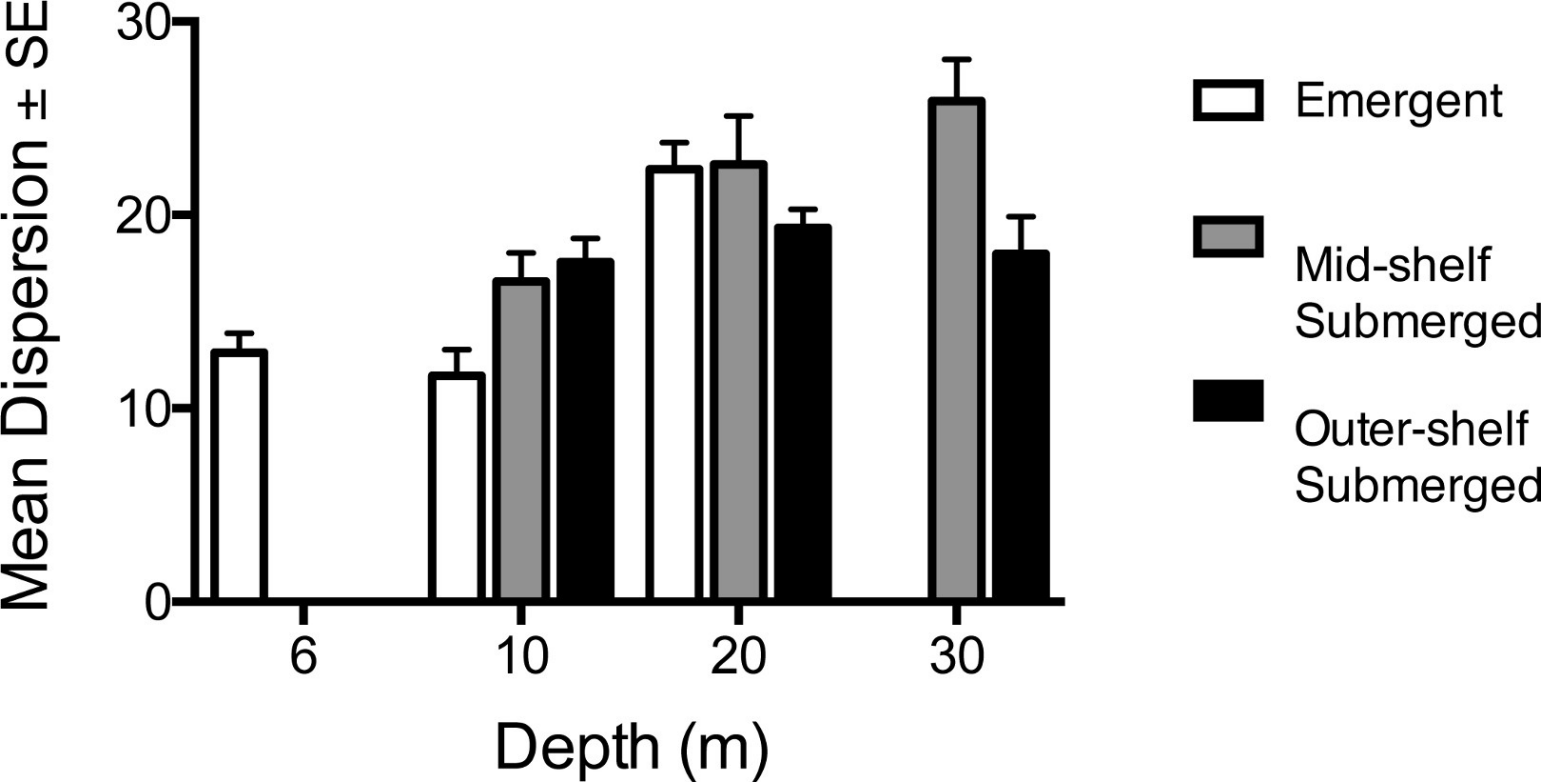
Differences in multivariate dispersion in the functional assemblage of the reef fish community among depths and reef types.

The functional structure of the reef fish community also varied among depths (*p* = 0.001) and among shelf positions (*p* = 0.001), with a significant interaction between the two factors (*p* = 0.01) (Table 1A). The PCO analysis indicated that 80% of variation in the fish community was explained by PCO axis 1, which closely followed the depth gradient (Fig 3A). This finding was supported by the BEST analysis, which indicated that depth alone was the best predictor of the fish assemblage (Rho = 0.267, *p* = 0.001). Spearman rank correlations indicated that mesopredators and soft coral/sponge feeding fishes were associated with deeper sites, but most other functional groups were associated with shallower sites (Fig 3B). PERMDISP also showed that similarity in the fish assemblages varied among depths and reef types (F_8,83_ = 4.21, *p* = 0.008), becoming more dissimilar with depth. However, dissimilarity at the deepest comparable depths was almost 30% lower on the outer-shelf (Fig 4). The functional composition therefore differed significantly (by up to 35% between 10 and 30 m) between all three depths on the mid-shelf reefs, but not between 20 m and 30 m depths on outer-shelf reefs. On emergent reefs the functional composition of the fish community was similar between the shallowest depths (6 m and 10 m) but differed from the community at 20 m, which was twofold greater than that at shallower depths (Table 2).

**Table 2:** Pairwise similarity of reef fish assemblages between reef types and depths identified using PERMANOVA analysis based on log-transformed functional group abundances.

	Em10	Em20	MS10	MS20	MS30	OS10	OS20	OS30
Em10	80.9							
Em20	64.6*	64.2						
MS10	74.1*	59.9***	75.6					
MS20	68.9	65.4	66.3***	66.9				
MS30	50.8***	62.0	46.2***	55.0**	62.2			
OS10	75.1	60.3***	73.8***	66.8***	46.0***	74.5		
OS20	73.2	67.5	67.8**	68.2*	53.3***	70.0***	72.1	
OS30	69.5**	70.0	64.0***	68.0	59.2***	65.8***	71.5	73.2

Em = Emergent, MS = Mid-shelf Submerged, OS = Outer-Shelf Submerged. Significant differences among reef types/depths are identified in bold. * indicates significant difference in community composition;

* = 0.05,

** = 0.01,

*** = 0.001.

The mean abundances of nine of the 11 functional groups varied significantly among depths, reef-types or a combination of the two. The mean abundance of algal feeders was most abundant at shallower depths. Both croppers and scrapers were significantly more abundant at 6–10 m (having a tenfold and sixfold increase compared to 30 m), while territorial farmers had a twofold increase in abundance at 10 m than at deeper depths. Both soft coral/sponge feeders and detritivores (after accounting for the reef effect) were more abundant on mid-shelf reefs than on outer-shelf reefs by 40–50% respectively, and detritivores were also about 30% more abundant at 30 m than at shallower depths. The means of three functional groups (benthic carnivores, site-attached planktivores (after accounting for reef effect), and omnivores) had complex distributions that responded to interactions among depth and reef type.

**Fig 5:**
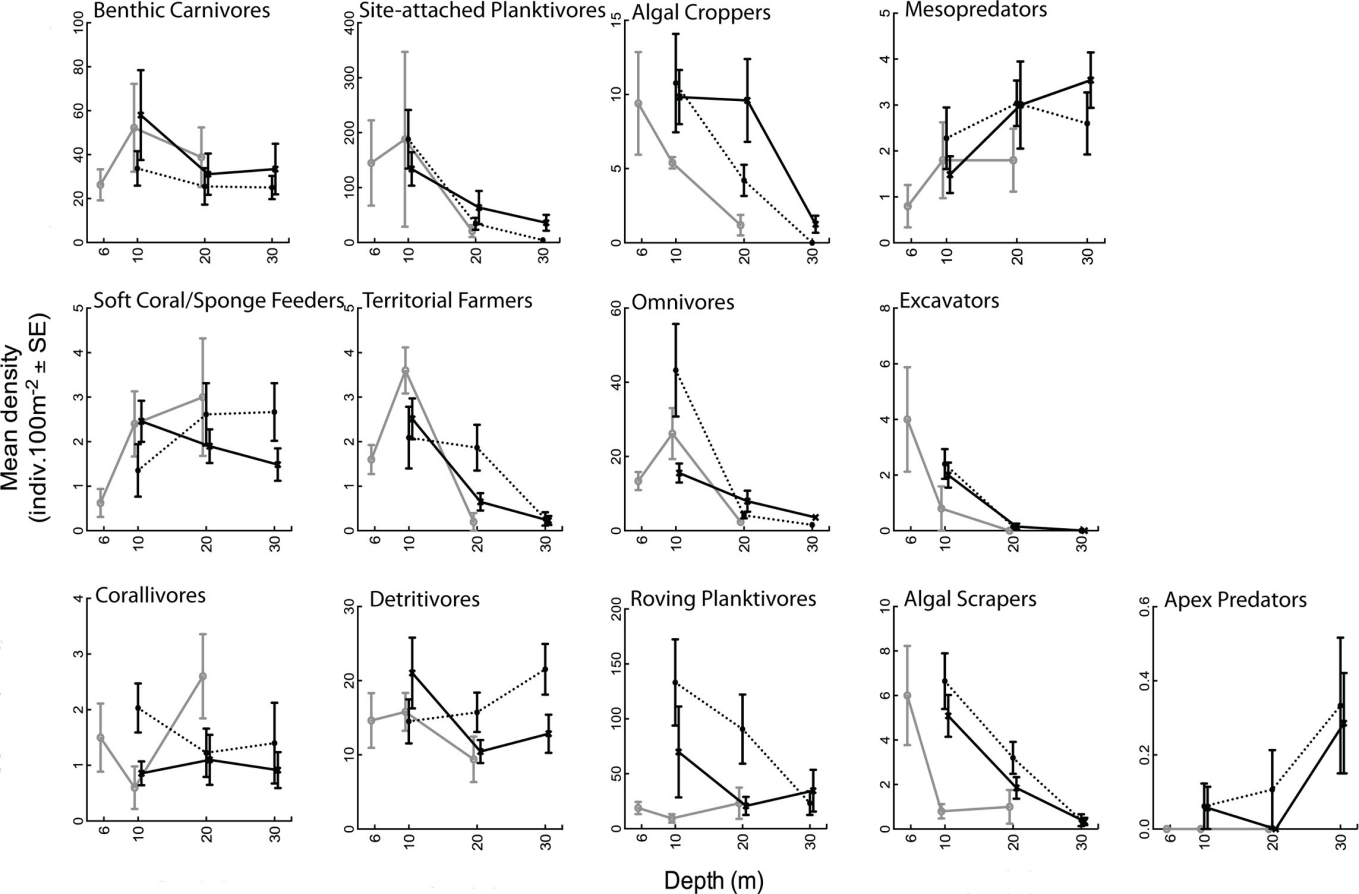
Mean fish abundance among depths and reef types for each functional group. Grey line = emergent reefs, dotted black line = mid-shelf submerged reefs, solid black line = outer-shelf submerged reefs.

Within depths, the functional structure of fish communities on emergent reefs at 10 m was similar to outer-shelf submerged reefs (t = 1.31, *p* = 0.12) but differed from mid-shelf submerged reefs (t = 1.67, *p* = 0.02). The mid-shelf submerged reefs supported around a twofold increase in the mean abundance of roving planktivores, omnivores and algal scrapers (Fig 5). At both 20 and 30 m, community composition was similar between emergent and submerged reefs (Em~MS t = 0.96, *p* = 0.48; Em~OS t = 1.21, *p* = 0.21), but different between mid-shelf and outer-shelf submerged reefs (20 m t = 1.50, *p* = 0.04, 30 m t = 2.49, *p* = 0.001). Differences among mid-shelf and outer-shelf submerged reefs were due to variability in the mean abundance of roving planktivores and soft coral/sponge feeders at 20 m, and site-attached planktivores, benthic carnivores, detritivores and soft coral/sponge feeders at 30 m (Fig 5).

### Relationships between benthic structure, fish distributions and functional composition

#### Total fish abundance & benthic structure

20% of variation in total fish abundance could be explained by changes in the availability of complex coral alone, and the addition of complex coral cover improved models of overall fish distributions by 13 AIC points (Table 1). However, there was no relationship between total fish abundance and the cover of either turf algae or soft corals and sponges (all comparisons *p* >0.05).

#### Functional composition & benthic structure

Changes in the functional assemblage of the reef fish community were best predicted by depth alone (Rho = 0.267, *p* = 0.001) and were not positively influenced by the addition of benthic predictors. As with total fish abundance, no functional group mean abundances were significantly related to changes in the availability of turf algae or soft corals and sponges (all comparisons *p* > 0.05). However, the mean abundances of seven functional groups were significantly related to changes in complex coral cover (Table 1), with the greatest effect on site-attached planktivores (R^2^ = 0.20). Even for these seven groups, complex coral explained a relatively small proportion of the total variation in abundance (r^2^ = 0.06–0.20). Nonetheless, taking into account heterogeneity in the distribution of complex coral cover by adding complex coral as an explanatory variable to the depth x reef type interaction improved model performance for four functional groups: benthic carnivores, territorial farmers, site-attached planktivores, algal croppers, as well as total fish abundance (Table 1). AIC scores differed by more than 10 points for models of site-attached planktivore and algal cropper distribution, and between 2 and 4 points for distribution models of benthic carnivores and territorial algal farmers.

## Discussion

Our results confirm that submerged reefs in the central GBR support abundant fish communities. As expected, mean total fish abundance and the mean abundance of most functional groups declined with depth [5, 34, 54], but abundance declined less steeply (twofold) on the outer-shelf submerged reefs than on both mid-shelf submerged reefs (sevenfold) and emergent reefs. Our finding that declines in abundance are less steep on the outer shelf suggests that environmental factors correlated with depth, rather than depth *per se*, are the primary drivers of fish abundance. Many environmental variables co-vary with depth, including light, pressure, and wave energy. Outer-shelf reefs occur in clear water with higher light irradiance than inner-shelf or mid-shelf reefs at any given depth [55], and are also more likely to be exposed to higher wave energy [56, 57]. Greater light penetration would increase the depth where photosynthesis, and therefore algal and coral growth, is possible, which theoretically allows herbivorous and coral associated species to occur at greater depths in clear waters. However, food limitation does not appear to be a key factor limiting the depth range of herbivorous fishes [34]. Light irradiance may influence feeding capacity in some groups, such as roving planktivores, where feeding capacity increases with light [21], while other groups, such as site-attached planktivores, may respond more to changes in habitat complexity [58, 59]. Previous studies have also shown that herbivory rates are higher on reef crests than lower slopes or reef flats [33], suggesting water motion is a key driver of both algal growth and herbivory. Increased water movement at greater depths on outer-shelf reefs may also increase the abundance of herbivorous fish at greater depths on the outer-shelf. Experimental approaches that explicitly test for the effects of co-varying factors (e.g. Smallhorn-West et al. [60]), such as light, temperature, and habitat availability, are needed to better understand the mechanisms generating depth-abundance relationships among functional groups.

Our study found ten out of the 13 functional groups to be associated with shallow depths, being associated with the upper reef slope of emergent reefs or the ‘crests’ of submerged reefs. The tops of mid-shelf submerged reefs in particular supported a high abundance of roving planktivores. Submerged reefs are often exposed to strong currents since there is no reef flat to block current flows [61], providing an ideal habitat for planktivorous fishes. These findings are supported by observations by the authors (CM and TB) on other submerged reefs, where high fish abundance occurs on the crest of submerged reefs regardless of depth. These observations support the hypothesis that water flow is an important determinant of fish abundance as hydrodynamics determine the complexity of the benthos [38] and food availability [33].

Only two functional groups (mesopredators, and soft coral/sponge feeders) were more abundant at deeper sites. The twofold increase in abundance of soft coral/sponge feeders was likely due to the higher abundance of soft corals and sponges at deeper sites [38, 62]. The lack of a significant correlation between soft coral/sponge abundance and fishes that prey on them could be due to the spatial scale examined. Most soft coral feeders are relatively mobile with spatial ranges of up to tens of meters [9], rather than the scale examined here. Conversely, habitat associations for other small, site-attached groups may have been stronger if we were able to examine fish-habitat relationships at smaller spatial scales. In any case, our results suggest that deeper reefs may represent important habitat for ecologically significant but numerically rare functional groups.

Apex predators were not sufficiently abundant to establish a significant relationship with depth, likely because our surveys were not designed to survey apex predators, and a different survey design, such as the inclusion of ‘roving’ surveys specifically targeting groups with low numerical abundance such as apex predators (e.g. Bejarano et al. [63]), or the use of baited remote underwater video stations (BRUVs) [64] would provide greater information on the importance of deeper habitats for this group. Given that apex and mesopredators are groups that are often the most heavily targeted by fishing, it is important to conduct further studies on deep submerged reef habitats in order to provide accurate ecosystem assessments.

Despite the congruent patterns in depth distributions between fish and benthic communities, few functional groups showed significant relationships with specific habitat types after accounting for depth and cross-shelf variability. The only benthic component significantly correlated with any functional group was complex coral. Even though it was found to explain a relatively small proportion of the total variation in abundance, this association is not surprising as the complex habitats created by a diversity of stony coral morphologies offer reef fish and other organisms a source of food and shelter [22–24]. This result indicates that complex coral provides important habitats throughout the depth gradient, particularly for site-attached fishes with smaller body sizes such as territorial farmers, site-attached planktivores, benthic carnivores and algal croppers. Highly complex corals, such as those with tight branches, support smaller reef fish, as they provide a refuge from predation, but size limitations exclude larger bodied fish from using these highly complex habitats [65]. Functional groups composed of larger-bodied species are less dependent on small-scale habitat, as their distributions are likely influenced instead by factors operating at broader spatial scales [59]. While it is possible that sample size may have impacted the significance of fish-benthos relationships found in this study, the lack of significant relationships found is more likely an artifact of scale. Since the functional groups observed in this study utilize habitats at different scales it is likely that the lack of significance between some fish-benthos relationships may be due to scale as this study only observed these relationships at one scale.

The functional composition of the fish community also varied significantly among depths and reef types, with community dissimilarity generally increasing with depth. This finding supports previous benthic studies indicating that the depth zonation of reef organisms shifts downwards on submerged reefs [5,38]. Many reef fishes occur across relatively broad depth ranges [40, 41, 5], allowing them to occur on deeper submerged reefs where suitable habitat occurs. Interestingly, communities characteristic of intermediate depths on submerged reefs penetrated deeper on the outer-shelf than the mid-shelf, a pattern also observed for reef fishes in Kimbe Bay [5] and benthic communities on the same reefs examined here [38]. These patterns are likely attributed to the differences in the hydrodynamic environment among the mid- and outer-shelf [38].

Our results suggest that submerged reefs have higher total fish abundance and a similar or higher abundance of 11 functional groups at comparable depths of 10 and 20 meters compared to emergent reefs. This could be attributed to different factors, including differences in hydrodynamics or variation in benthic composition [38]. The variability in fish abundance among reef morphologies, even on nearby reefs, highlight the importance of considering changes in the abundance and composition of fish assemblages across habitat types to better understand the ecological dynamics and population trajectories of coral reef fishes. Previous studies describing GBR fish communities are based primarily on shallow data only. However, our study within the Cairns sector shows variance in fish communities among relatively small depth ranges (6–30 m) and therefore we recommend that caution should be used when comparing LTMP data to fish communities in general within this sector of the GBR.

Our study confirms that the ubiquitous submerged reefs of the central GBR support abundant fish assemblages, providing further evidence of their significance as an important component of the GBR ecosystem. In addition, we show that the abundance of ecologically important functional groups, and therefore key ecological processes such as herbivory, vary significantly along depth gradients. A greater understanding of whether and how these key processes vary with depth and among reef types will provide greater insight into the dynamics reefs in the GBR more broadly, including the capacity for deeper habitats to act as source reefs following disturbance [61]. Given the urgent need to understand factors such as connectivity among reefs for managing the GBR ecosystem under increasing stressors, we recommend that the extensive submerged reefs be given greater consideration when assessing the status and trajectory of the GBR ecosystem.

## Supporting information

S1 Table Allocation of fish species to functional group, geographical range, habitat and depth range. (PDF)

S2 Table Sampling design of the eight submerged reefs sampled in the Cairns region of the GBR, Australia. (PDF)

S3 Table Total abundance of each functional group across all transects on eight submerged reefs and two emergent reefs in the Cairns region of the GBR, Australia. Individuals were allocated to functional group using S1 Table. Transects on submerged reefs were conducted at 10, 20 and 30 m (+/- 2 m). Transects on emergent reefs were conducted at 6, 10 and 20 m (+/- 2 m).
(XLSX)
