# Supplementary material for: Variability in the functional composition of coral reef fish communities on submerged and emergent reefs in the central Great Barrier Reef, Australia
Source: PLoS One. 2019 May 17;14(5):e0216785. doi: 10.1371/journal.pone.0216785 (PMC6524821; doi:10.1371/journal.pone.0216785)
Supplement: S1 Table — (PDF) [file pone.0216785.s001.pdf]

Family	Species	Functional Group	Location	Habitat	Depth	Reference
**Acanthuridae**	*Acanthurus blochii*	Detritivore	E. Africa to Hawaii and Society Is. in French Polynesia – S.W. Japan to Great Barrier Reef and Lord Howe I.	Lagoon and outer reef	2 – 15 m	Allen et al., 2003; Williamson et al., 2014
	Acanthurus dussumieri	Detritivore	E. Africa to Hawaii and Line Is. in eastern Central Pacific – S.W. Japan to Great Barrier Reef and Lord Howe I.	Seaward reefs	9 – 131 m	Allen et al., 2003; Williamson et al., 2014
	Acanthurus grammoptilus	Detritivore	Indonesia, Philippines, Papua New Guinea, Solomon Is. and N. Australia	Inshore reefs	2 – 20 m	Allen et al., 2003; Williamson et al., 2014
	Acanthurus lineatus	Algal cropper	E. Africa to Indonesia, Micronesia and Polynesia – S.W. Japan to Great Barrier Reef and New Caledonia	Outer edge of reefs	0 – 6 m	Allen et al., 2003; Williamson et al., 2014
	Acanthurus nigricauda	Detritivore	E. Africa to French Polynesia – S.W. Japan to Great Barrier Reef	Over sand near coral or rock outcrops	3 – 30 m	Allen et al., 2003; Williamson et al., 2014
	Acanthurus nigrofuscus	Algal cropper	Red Sea and E. Africa to Micronesia, Hawaii and Polynesia – S.W. Japan to Great Barrier Reef and Lord Howe I.	Inshore and outer reefs	0 – 20 m	Allen et al., 2003; Williamson et al., 2014
	Acanthurus xanthopterus	Detritivore	E. Africa to Mexico – S.W. Japan to Great Barrier Reef and New Caledonia	Sandy areas near reefs	15 – 90 m	Allen et al., 2003; Williamson et al., 2014
	Ctenochaetus binotatus	Detritivore	E. Africa to Indonesia, Micronesia and French Polynesia – S.W. Japan to Great Barrier Reef	Rubble areas of lagoons and seaward reefs	12 – 53 m	Allen et al., 2003; Williamson et al., 2014
	Ctenochaetus striatus	Detritivore	E. Africa to Indonesia, Micronesia and French Polynesia – S.W. Japan to Great Barrier Reef	Lagoon and seaward reefs	0 – 35 m	Allen et al., 2003; Williamson et al., 2014
	Naso annulatus	Algal cropper	E. Africa to Indonesia, Micronesia, Hawaii and Polynesia – S.W. Japan to Great Barrier Reef and Lord Howe I.	Steep outer reef slopes	20 – 60	Allen et al., 2003; Williamson et al., 2014
	Naso brevirostris	Algal cropper	Red Sea and E. Africa to Galapagos Is. off Ecuador – S.W. Japan to Great Barrier Reef and Lord Howe I.	Lagoon and seaward reefs	4 – 46 m	Allen et al., 2003; Williamson et al., 2014
	Naso lituratus	Algal cropper	Red Sea and E. Africa to Hawaii, Pitcairn Is. – S.W. Japan to S. Great Barrier Reef and New Caledonia	Lagoon and outer reefs	0 – 70 m	Allen et al., 2003; Williamson et al., 2014
	Naso unicornis	Algal cropper	Red Sea and E. Africa to Micronesia, Hawaii and French Polynesia – S.W. Japan to Great Barrier Reef and Lord Howe I.	Lagoons and outer reefs	1 – 80 m	Allen et al., 2003; Williamson et al., 2014
	Zebrasoma scopas	Algal cropper	E. Africa to Indonesia, Philippines and French Polynesia – S.W. Japan to Great Barrier Reef and Lord Howe I.	Lagoons and outer reefs	0 – 50 m	Allen et al., 2003; Williamson et al., 2014
	Zebrasoma veliferum	Algal cropper	Indonesia, Micronesia, Hawaii to French Polynesia – S.W. Japan to Great Barrier Reef and New Caledonia	Lagoon and outer reefs	0 – 45 m	Allen et al., 2003; Williamson et al., 2014
**Anthiinae**	*Luzonichthys waitei*	Planktivore	Aldabra to New Caledonia and Fiji – S.W. Japan to Great Barrier Reef	Steep outer reef slopes	0 – 35	Allen et al., 2003
	Plectanthias longimanus	Planktivore	E. Africa to Fiji – S.W. Japan to Great Barrier Reef	Crevices and ledges of reefs	6 – 73 m	Allen et al., 2003
	Plectanthias winniensis	Planktivore	Red Sea to Hawaii and Pitcairn Is. east of French Polynesia – Marshall Is. in Micronesia to Great Barrier Reef and New Caledonia	Outer reef slopes	23 – 58 m	Allen et al., 2003
	Pseudanthias bicolour	Planktivore	Mauritius to Hawaii and Line Is. in eastern Central Pacific – S.W. Japan to Australia	Ledges and outcroppings of lagoons and outer reefs	5 – 68 m	Allen et al., 2003
	Pseudanthias cooperi	Planktivore	E. Africa to Samoa and Line Is. in eastern Central Pacific – S.W. Japan to Micronesia and Australia	Outer reefs and drop-offs	15 – 60 m	Allen et al., 2003
	Pseudanthias dispar	Planktivore	Christmas I. to Line Is. in eastern Central Pacific – S.W. Japan and Micronesia to Great Barrier Reef	Upper edge of steep slopes	0 – 15 m	Allen et al., 2003
	Pseudanthias engelhardi	Planktivore	Great Barrier Reef and E. Papua New Guinea to Fiji	Outer reefs	50 – 70 m	Allen et al., 2003
	Pseudanthias fasciatus	Planktivore	Red Sea to Papua New Guinea – S.W. Japan to Palau and Great Barrier Reef	Caves of deep outer reefs and steep slopes	20 – 68 m	Allen et al., 2003
	Pseudanthias huchti	Planktivore	Indonesia, Philippines, Palau, Papua New Guinea, Solomon Is., Vanuatu and Great Barrier Reef	Outer reef slopes	4 – 20 m	Allen et al., 2003
	Pseudanthias hypselosoma	Planktivore	Maldives to Samoa – S.W. Japan to Palau and Great Barrier Reef	Sheltered coastal reefs and lagoons	0 – 35 m	Allen et al., 2003
	Pseudanthias lori	Planktivore	Christmas I. to Philippines, Fiji and French Polynesia – S.W. Japan to N.W. Australia and Great Barrier Reef	Steep outer reef slopes and drop-offs	25 – 60 m	Allen et al., 2003
	Pseudanthias luzonensis	Planktivore	Indonesia and Philippines to Fiji – Taiwan to Palau and Great Barrier Reef	Outer reef slopes	20 – 60 m	Allen et al., 2003
	Pseudanthias pascalus	Planktivore	N. Sulawesi in Indonesia and Micronesia to French Polynesia – Taiwan and S.W. Japan to Great Barrier Reef	Outer reef slopes	5 – 60 m	Allen et al., 2003
	Pseudanthias pictilis	Planktivore	S. Great Barrier Reef, New Caledonia and Lord Howe I.	Seaward reef slopes	20 – 40 m	Allen et al., 2003
	Pseudanthias pleurotaenia	Planktivore	Indonesia to Samoa – S.W. Japan and Micronesia to N.W. Australia and New Caledonia	Steep seaward slopes	10 – 180 m	Allen et al., 2003
	Pseudanthias rubrizonatus	Planktivore	Andaman Sea to Solomon Is. – S.W. Japan to Palau, N.W. Australia and Great Barrier Reef	Coastal and seaward reefs	10 – 58 m	Allen et al., 2003
	Pseudanthias sheni	Planktivore	N.W. Australia	Offshore reefs	20 – 55 m	Allen et al., 2003
	Pseudanthias smithvanizi	Planktivore	Cocos-Keeling Is. to Palau and Marshall Is. in Micronesia – S.W. Japan to Great Barrier Reef	Steep outer reef slopes	6 – 70 m	Allen et al., 2003
	Pseudanthias squamipinnis	Planktivore	Red Sea and E. Africa to Solomon Is. and Fiji – S.W. Japan and Palau and E. Africa	Coastal, lagoon and outer reefs	2 – 20 m	Allen et al., 2003
	Pseudanthias tuka	Planktivore	Indonesia to Solomon Is. – S.W. Japan, Palau to N.W. Australia, Great Barrier Reef, New Caledonia and Vanuatu	Outer reef slopes and coastal reefs	2 – 40 m	Allen et al., 2003
	Pseudanthias ventralis	Planktivore	Great Barrier Reef to Pitcairn I. east of French Polynesia, north to Mariana Is. in Micronesia	Outer reefs	26-68 m	Allen et al., 2003
	Serranocirrhitus latus	Planktivore	Indonesia to Fiji – S.W. Japan to Palau, Great Barrier Reef and New Caledonia	Under ledger or near recesses in outer reef drop-offs	15 – 70 m	Allen et al., 2003
**Caesionodae**	*Caesio caerulaurea*	Planktivore	Red Sea and E. Africa to Samoa – S.W. Japan to Micronesia and Great Barrier Reef	Upper edge of steep slopes and around	0 – 30 m	Allen et al., 2003

				coastal patch reefs and seaward reefs		
	Caesio cunning	Planktivore	E. India and Sri Lanka to Vanuatu. – S.W. Japan to Palau, N.W. Australia and Great Barrier Reef	Upper edge of steep slopes and around coastal patch reefs	0 – 30 m	Allen et al., 2003
	Caesio lunaris	Planktivore	Red Sea and. Africa to Solomon Is and Fiji – S.W. Japan to Palau and Great Barrier Reef	Upper edge of steep slopes, coastal patch and seaward reefs	0 – 30 m	Allen et al., 2003
	Caesio teres	Planktivore	E. Africa to Micronesia, Samoa and Line Is. in eastern Central Pacific – S.W. Japan to Great Barrier Reef	Upper edge of steep slopes, around coastal patch reefs and seaward reefs	0 – 30 m	Allen et al., 2003
	Dipterygonatus balteatus	Planktivore	Gulf of Aden and Somalia to Solomon Is. – Taiwan to Australia	Coastal, lagoons and seaward reefs	0 – 20 m	Allen et al., 2003
	Pterocaesio chrysozona	Planktivore	Red Sea and E. Africa to Papua New Guinea – S.W. Japan to Australia	Coastal, lagoons and other reefs	2 – 25 m	Allen et al., 2003
	Pterocaesio digramma	Planktivore	E. Malaysian Peninsula and S.W. Japan to Australia, Fiji and New Caledonia	Steep slopes, patch reefs and seaward reefs	0 – 30 m	Allen et al., 2003
	Pterocaesio pisang	Planktivore	E. Africa to reefs offshore N.W Australia, Philippines and Fiji	Steep slopes and shallow coastal and seaward reefs	0 – 30 m	Allen et al., 2003
	Pterocaesio trilineata	Planktivore	E. Africa to Fiji, Line Is. and French Polynesia – S.W. Japan to E. Africa	Steep slopes, shallow coastal, lagoon and seaward reefs	0 – 30 m	Allen et al., 2003
**Carcharhinidae**	*Carcharhinus albimarginatus*	Apex predator	Red Sea and E. Africa to Society Is. in French Polynesia – S. Japan to Australia	Outer reef slopes	Below 20 m	Allen et al., 2003
	Carcharhinus amblyrhynchos	Apex predator	Madagascar and Seychelles to Hawaii and Pitcairn I. east of French Polynesia – China to Australia	Outer reef slopes	1 – 274 m	Allen et al., 2003
	Carcharhinus falciformis	Apex predator	Circumtropical	Open waters	0 – 500 m	Allen et al., 2003
	Carcharhinus galapagensis	Apex predator	Tropical and warm temperate seas	Around islands	2 – 180 m	Allen et al., 2003
	Carcharhinus leucas	Apex predator	Tropical and warm temperate seas	Coastal reefs and estuaries	0 – 152 m	Allen et al., 2003
	Carcharhinus limbatus	Apex predator	Tropical and subtropical seas	Lagoons, inshore waters and reef channels	0 – 50 m	Allen et al., 2003
	Carcharhinus melanopterus	Apex predator	E. Africa and Red Sea to Hawaii and Pitcairn I. east of French Polynesia – S. Japan to Australia	Coastal, lagoon and outer slopes	1 – 20 m	Allen et al., 2003
	Carcharhinus obscurus	Apex predator	Circumtropical	Continental coastlines	0 – 400 m	Allen et al., 2003
	Carcharhinus plumbeus	Apex predator	Tropical and subtropical seas	Over sand or mud, occasionally near reefs	0 – 280 m	Allen et al., 2003
	Galeocerdo cuvier	Apex predator	Tropical and temperate seas	Coastal and offshore reefs	0 – 75 m	Allen et al., 2003
	Negaprion acutidens	Apex predator	E. Africa and Red Sea to Society IS. in French Polynesia – Marshall Is. in Micronesia to Australia	Bottom of bays, estuaries and offshore reefs	1 – 30 m	Allen et al., 2003
	Triaenodon obesus	Apex predator	E. Africa and Red Sea to Hawaii and E. Pacific – S. Japan to Australia	Coastal, lagoon and outer reef slopes	3 – 122 m	Allen et al., 2003
**Chaetodontidae**	*Chaetodon aureofasciatus*	Corallivore	N. Australia and S. Papua New Guinea to Great Barrier Reef	Silty coastal reefs, often near river mouths	5 – 15 m	Allen et al., 2003; Williamson et al., 2014
	Chaetodon auriga	Corallivore	Red Sea and E. Africa to Hawaii and French Polynesia – S.W. Japan to E. Australia and Lord Howe I.	Coastal and outer reefs	0 – 40m	Allen et al., 2003; Williamson et al., 2014
	Chaetodon baronessa	Corallivore	Cocos-Keeling Is. to Philippines and Fiji – S.W. Japan and Micronesia to Great Barrier Reef	Near *Acropora* plate corals	0 – 10m	Allen et al., 2003; Williamson et al., 2014
	Chaetodon citrinellus	Corallivore	E. Africa to Indonesia, Philippines and Marquesas Is. in French Polynesia – S.W. Japan to N.W. & E. Australia	Moderately exposed (to surf) reef flats and seaward reefs	1 – 3 m; rarely to 30 m	Allen et al., 2003; Williamson et al., 2014
	Chaetodon flavirostris	Corallivore	E. Australia to Rapa and Pitcairn Is. south east of French Polynesia	Coral and rocky reefs	2 – 20 m	Allen et al., 2003; Williamson et al., 2014
	Chaetodon lineolatus	Corallivore	E. Africa, Red Sea, Maldives to Hawaii and French Polynesia – S.W. Japan to N.W. & E. Australia	Lagoons and seaward reefs from shallows	0 – 171 m	Allen et al., 2003; Williamson et al., 2014
	Chaetodon lunula	Corallivore	E. Africa to Indonesia, Micronesia, Hawaii and Galapagos – S.W. Japan to N.W. & E. Australia	Lagoons and outer reefs	0 – 30 m	Allen et al., 2003; Williamson et al., 2014
	Chaetodon lunulatus	Benthic carnivore	Indonesia, Malaysian Peninsula, Philippines, New Guinea, Hawaii to French Polynesia – S.W. Japan to N.W. Australia and Great Barrier Reef	Coral-rich areas	0 – 20m	Allen et al., 2003; Williamson et al., 2014
	Chaetodon melannotus	Benthic carnivore	Red Sea and E. Africa to Indonesia, New Guinea and Samoa – S.W. Japan and Philippines to E. Australia	Coral-rich areas of lagoons, reef flats and seaward reefs	2 – 20 m	Allen et al., 2003; Williamson et al., 2014
	Chaetodon ornatissimus	Corallivore	Maldives to Indonesia, Philippines, Micronesia and French Polynesia – S.W. Japan to N.W. & N.E. Australia	Coral-rich areas in clear water lagoons and seaward reefs	0 – 36 m	Allen et al., 2003; Williamson et al., 2014
	Chaetodon plebeius	Corallivore	Andaman Sea to Indonesia, New Guinea and Fiji – S.W. Japan to W. & E. Australia	Shallow coastal waters including lagoons and seaward reefs	0 – 10 m	Allen et al., 2003; Williamson et al., 2014
	Chaetodon rafflesi	Corallivore	Sri Lanka to Indonesia, New Guinea and French Polynesia – S.W. Japan to Great Barrier Reef	Coral-rich areas of sheltered coastal reefs, lagoons and outer slopes	0 – 15 m	Allen et al., 2003; Williamson et al., 2014
	Chaetodon rainfordi	Corallivore	S. Papua New Guinea to Great Barrier Reef	Coastal and offshore reef areas of sparse coral growth	0 – 15 m	Allen et al., 2003; Williamson et al., 2014
	Chaetodon speculum	Corallivore	Indonesia, Malaysian Peninsula, Philippines, New Guinea to Tonga - S.W. Japan to N.W. & E. Australia	Coral-rich reefs in lagoons and on outer reefs	8 – 30 m	Allen et al., 2003; Williamson et al., 2014
	Chaetodon trifascialis	Corallivore	Red Sea and E. Africa to Indonesia, Philippines, Micronesia, Hawaii and French Polynesia – S.W. Japan to N.E. & E. Australia	Coral-rich areas	0 – 12m	Allen et al., 2003; Williamson et al., 2014
	Chaetodon ulietensis	Benthic carnivore	Cocos-Keeling Is. to Indonesia, Malaysian Peninsula, Philippines, Micronesia and French Polynesia – S.W. Japan to E. Australia	Coral-rich areas of lagoons and seaward	0 – 30 m	Allen et al., 2003; Williamson et al.,

				reefs		2014
	Chaetodon vagabundus	Corallivore	E. Africa to Hawaii and French Polynesia – S.W. Japan to E. Australia and Lord Howe I.	Coastal reef and outer slopes	0 - 30m	Allen et al., 2003; Williamson et al., 2014
	Chelmon rostratus	Benthic carnivore	E. Andaman Sea to Indonesia, Philippines to Solomon Is. – S.W. Japan to E. Australia	Coastal, inner reefs and estuaries, often in turbid water	0 – 25 m	Allen et al., 2003; Williamson et al., 2014
	Coradion altivelis	Benthic carnivore	Andaman Sea to Indonesia, Philippines, New Guinea and Solomon Is. – S.W. Japan to E. Australia	Inshore reefs	3 – 15 m	Allen et al., 2003; Williamson et al., 2014
	Coradion chrysostomus	Benthic carnivore	Andaman Sea to Indonesia, Philippines, New Guinea and Solomon Is. – S.W. Japan to N. & E. Australia	Coastal reefs	3 – 60 m	Allen et al., 2003; Williamson et al., 2014
	Heniochus acuminatus	Benthic carnivore	Red Sea and E. Africa to Indonesia, Philippines and French Polynesia – S.W. Japan to W. & E. Australia	Lagoons and outer reef slopes	2 – 75 m	Allen et al., 2003; Williamson et al., 2014
	Heniochus monoceros	Benthic carnivore	E. Africa, Bay of Bengal to Indonesia, Philippines, New Guinea and French Polynesia – S.W. Japan to E. Australia	Lagoons and outer reefs with rich coral growth	2 – 25 m	Allen et al., 2003; Williamson et al., 2014
	Heniochus varius	Benthic carnivore	Indonesia, Philippines, Micronesia and Fiji to Polynesia – S.W. Japan to N.W. & N.E. Australia and New Caledonia	Coral-rich areas of lagoons and seaward reef slopes	2 – 30 m	Allen et al., 2003; Williamson et al., 2014
	Parachaetodon ocellatus	Benthic carnivore	Indonesia to Philippines, New Guinea and Fiji – S. China Sea and S.W. Japan to N.W. & E. Australia	Coastal and inner reefs littered with sponges	5 – 40 m	Allen et al., 2003; Williamson et al., 2014
**Ephippidae**	*Platax orbicularis*	Benthic carnivore	Red Sea and E. Africa to Indonesia, Micronesia and Polynesia – S.W. Japan to N.E. Australia and New Caledonia	Shoreline and outer reefs	2 – 35 m	Allen et al., 2003; Williamson et al., 2014
	Platax pinnatus	Benthic carnivore	Sumatra in Indonesia to Solomon Is., Vanuatu and Fiji – S.W. Japan to N. Australia and New Caledonia	Coastal reefs and seaward slopes	2 – 25 m	Allen et al., 2003; Williamson et al., 2014
	Platax teira	Benthic carnivore	Red Sea and E. Africa to Solomon Is. and Fiji – S.W. Japan to Great Barrier Reef	Inshore and outer reefs	3 – 25 m	Allen et al., 2003; Williamson et al., 2014
**Ginglymostomatidae**	*Nebrius ferrugineus*	Apex predator	Red Sea and E. Africa to Society Is. in French Polynesia – S. Japan to Australia	Lagoon and seaward reefs	1 – 70 m	Allen et al., 2003
**Haemulidae**	*Diagramma pictum*	Mesopredator	Red Sea and E. Africa to Vanuatu and Fiji – S.W. Japan to Australia and New Caledonia	Sand bottoms of coastal and lagoon reefs	5 – 40 m	Allen et al., 2003; Williamson et al., 2014
	Plectorhinchus chaetodontoides	Mesopredator	Maldives to Fiji – S.W. Japan to Australia	Ledges of coastal reefs, lagoons and seaward reefs	2 – 30 m	Allen et al., 2003; Williamson et al., 2014
	Plectorhinchus flavomaculatus	Mesopredator	Red Sea and E. Africa to Papua New Guinea – S.W. Japan to Australia	Sheltered coastal reefs and lagoons	2 – 25 m	Allen et al., 2003; Williamson et al., 2014
	Plectorhinchus gibbosus	Mesopredator	Red Sea and E. Africa to Samoa – S.W. Japan to Australia	Coastal reefs, lagoons and outer reef slopes	0 – 25 m	Allen et al., 2003; Williamson et al., 2014
	Plectorhinchus lessonii	Mesopredator	Malaysian Peninsula and S.W. Japan to Great Barrier Reef and New Caledonia	Coastal reefs, lagoons and seaward reefs	0 – 35 m	Allen et al., 2003; Williamson et al., 2014
	Plectorhinchus unicolor	Mesopredator	Papua New Guinea to Queensland and the Great arrier Reef	Sheltered areas of reefs	N/A	Allen et al., 2003; Williamson et al., 2014
**Hemiscyllidae**	*Hemiscylliium ocellatum*	Apex predator	Queensland and N. Australia to New Guinea	Bottom of reefs	0 – 10 m	Allen et al., 2003
	Hemiscylliium trispeculare	Apex predator	Northern half of Australia	Sheltered reefs	0 – 4 m	Allen et al., 2003
**Kyphosidae**	*Kyphosus spp.*	Algal cropper	Red Sea and E. Africa to Line Is. and French Polynesia – S.W. Japan	Rocky shores, reef flats, lagoons, outer and seaward reefs	0 – 25 m	Allen et al., 2003; Williamson et al., 2014
	Microcanthus strigatus	Benthic carnivore	Isolated populations in E. and W. Australia, N. New Caledonia, Taiwan, S.W. Japan and Hawaii	Lagoons, rocky areas and shallow coral reefs	N/A	Allen et al., 2003; Williamson et al., 2014
**Labridae**	*Anampses geographicus*	Benthic carnivore	Mauritius and S.W. Australia to Caroline Is. in Micronesia and Fiji – S.W. Japan to S.E. Australia	Mix with algae and soft corals on reef tops and reef slopes	0 – 25 m	Allen et al., 2003; Williamson et al., 2014
	Bodianus axillaris	Benthic carnivore	Red Sea and E. Africa to Pitcairn Is. east of French Polynesia – S. Japan to Australia	Clear water lagoons and outer reefs	2 – 40 m	Allen et al., 2003; Williamson et al., 2014
	Cheilinus chlorurus	Benthic carnivore	E. Africa to Micronesia and Tuamotu, Marquesas and Rapa Is. in French Polynesia – S.W. Japan to E. Australia	Mixed sand, coral and rubble areas of lagoons and coastal reefs	2 – 30 m	Allen et al., 2003; Williamson et al., 2014
	Cheilinus fasciatus	Benthic carnivore	Red Sea to Micronesia and Samoa – S.W. Japan to E. Australia and New Caledonia	Mixed sand, coral and rubble areas of lagoons and outer reefs	3 – 40 m	Allen et al., 2003; Williamson et al., 2014
	Cheilinus trilobatus	Benthic carnivore	E. Africa to Micronesia and Tuamotu Is. in French Polynesia – S.W. Japan to Australia and New Caledonia	Lagoons, passes and outer reefs	0 – 30 m	Allen et al., 2003; Williamson et al., 2014
	Cheilinus undulatus	Benthic carnivore	Red Sea and E. Africa to Micronesia and Tuamotu Is. in French Polynesia – S.W. Japan to Australia and New Caledonia	Lagoon and outer reefs	0 – 60 m	Allen et al., 2003; Williamson et al., 2014
	Choerodon anchorago	Benthic carnivore	India to Palau and Yap in Micronesia and New Guinea – S.W. Japan to Australia and New Caledonia	Mixed seagrass, sand and rubble and coral areas of coastal reefs, shallow flats and lagoons	0 – 25 m	Allen et al., 2003; Williamson et al., 2014
	Choerodon cyanodus	Benthic carnivore	Sri Lanka to Papua New Guinea – S.W. Japan to Australia and New Caledonia	Sand and rubble area of coastal reefs	2 – 35 m	Allen et al., 2003; Williamson et al., 2014
	Choerodon fasciatus	Benthic carnivore	Two separate ranges – Taiwan and S.W. Japan to Palau. Also Great Barrier Reef and New Caledonia	Coastal outer reefs	0 – 15 m	Allen et al., 2003; Williamson et al., 2014
	Choerodon graphicus	Corallivore	Great Barrier Reef and New Caledonia	Sand and rubble patches of coastal lagoon and seaward reefs	2 – 30 m	Allen et al., 2003: Cole et al., 2008
	Choerodon monostigma	Benthic carnivore	N. Australia and New Guinea	Sand and weedy rubble areas	10 – 40 m	Allen et al., 2003; Williamson et al., 2014

	Choerodon schoenleinii	Benthic carnivore	Indonesia to Papua New Guinea – S.W. Japan to N. Australia	Sand, rubble and weedy areas on flat bottoms of lagoon and seaward reefs	10 – 60 m	Allen et al., 2003; Williamson et al., 2014
	Choerodon vitta	Benthic carnivore	Aru Is. in Indonesia, New Guinea and N. Australia	Flat sand and rubble bottoms near coastal reefs	10 – 40 m	Allen et al., 2003; Williamson et al., 2014
	Diproctacanthus xanthurus	Corallivore	Indonesia, Philippines to Solomon Is. and Great Barrier Reef	Sheltered reefs	3 – 25 m	Allen et al., 2003: Cole et al., 2008
	Epibulus insidiator	Benthic carnivore	Red Sea and E. Africa to Micronesia, Hawaii and Tuamotu Is. in French Polynesia – S. Japan to Australia	Coral-rich areas of lagoons and outer seaward reefs	0 – 42 m	Allen et al., 2003; Williamson et al., 2014
	Gomphosus varius	Benthic carnivore	Indonesia to Hawaii, Marquesas and Rapa Is. in French Polynesia – S. Japan to Australia	Coral-rich areas of lagoons and seaward reefs	0 – 35 m	Allen et al., 2003; Williamson et al., 2014
	Halichoeres melanurus	Benthic carnivore	Red Sea and E. Africa to Micronesia and French Polynesia – S.W. Japan to S.E. Australia and Lord Howe I.	Mixed sand, rubble and coral areas	0 – 30 m	Allen et al., 2003; Williamson et al., 2014
	Hemigymnus fasciatus	Benthic carnivore	Red Sea and E. Africa to Micronesia, Line Is. and Duie I. east of French Polynesia	Mixed sand, rubble and coral areas of lagoons, passes and outer slopes	0 – 25 m	Allen et al., 2003; Williamson et al., 2014
	Hemigymnus melapterus	Benthic carnivore	Indonesia, Micronesia to Samoa – S.W. Japan to Great Barrier Reef	Sheltered reefs	0 – 15 m	Allen et al., 2003; Williamson et al., 2014
	Labrichthys unilineatus	Corallivore	E. Africa to Samoa – S.W. Japan to Australia	Sheltered reefs with rich coral growth	0 – 20 m	Allen et al., 2003: Cole et al., 2008
	Labroides bicolor	Benthic carnivore	E. Africa to Micronesia, Line Is. and French Polynesia – S. Japan to E. Australia and Lord Howe I.	Coral reefs	2 – 25 m	Allen et al., 2003; Williamson et al., 2014
	Labropsis alleni	Corallivore	Indonesia and Philippines to Solomon Is. and Marshall Is. in Micronesia	Steep slopes of lagoon and seaward reefs	4 – 52 m	Allen et al., 2003: Cole et al., 2008
	Labropsis australis	Corallivore	Great Barrier Reef and Solomon Is. to Fiji and Samoa	Coral-rich areas of lagoons, outer reefs and passes	2 – 55 m	Allen et al., 2003: Cole et al., 2008
	Labropsis manabei	Corallivore	Hibernia Reef in Timor Sea, E. Indonesia and Papua New Guinea, north to S.W. Japan	Coral-rich areas	15 – 30 m	Allen et al., 2003: Cole et al., 2008
	Labropsis xanthonota	Corallivore	E. Africa to Samoa – S.W. Japan to Great Barrier Reef	Coral-rich areas of clear lagoons to seaward reefs	7 – 55 m	Allen et al., 2003: Cole et al., 2008
	Oxycheilinus diagramma	Benthic carnivore	Red Sea and E. Africa to Micronesia, Samoa and Fiji – S.W. Japan to New Caledonia	Coral-rich lagoons and seaward reefs	3 – 60 m	Allen et al., 2003; Williamson et al., 2014
	Stethojulis bandanensis	Benthic carnivore	E. Andaman Sea and W. Australia to Tuamotu Is. in French Polynesia – S. Japan to E. Australia	Reef flats and coastal shallows	0 – 20 m	Allen et al., 2003; Williamson et al., 2014
	Thalassoma hardwicke	Benthic carnivore	E. Africa to Line Is., Australia and Tuamotu Is. in French Polynesia – S. Japan to E. Australia	Coastal, lagoon and outer reefs	0 – 15 m	Allen et al., 2003; Williamson et al., 2014
**Lethrinidae**	*Gymnocranius spp.*	Mesopredator	S.W. Japan to N. Papua New Guinea, Great Barrier Reef, Coral Sea and New Caledonia	Open sand or rubble bottom of coastal reefs, lagoons and outer slopes	5 – 50 m	Allen et al., 2003; Williamson et al., 2014
	Lethrinus atkinsoni	Mesopredator	Indonesia to Tuamotu Is. in French Polynesia – S.W. Japan to Great Barrier Reef and Lord Howe I.	Outer reef slopes, sandy areas of lagoons and sea grass beds	2 – 25 m	Allen et al., 2003; Williamson et al., 2014
	Lethrinus laticaudis	Mesopredator	Lesser Sunda Is. in Indonesia, N. Australia, Papua New Guinea to Solomon Is.	Sandy areas near reef slopes	5 – 35 m	Allen et al., 2003; Williamson et al., 2014
	Lethrinus lentjan	Mesopredator	Red Sea and E. Africa to Tonga – S.W. Japan to N. Australia and New Caledonia	Sandy areas and coastal reefs, lagoons and outer slopes	10 – 50 m	Allen et al., 2003; Williamson et al., 2014
	Lethrinus miniatus	Mesopredator	N. Australia, Coral Sea and New Caledonia	Sand and Rubble	5 – 35 m	Allen et al., 2003; Williamson et al., 2014
	Lethrinus nebulosus	Mesopredator	Red Sea and E. Africa to Samoa – S.W. Japan to Australia	Flat sand bottoms in the vicinity of reefs	0 – 75 m	Allen et al., 2003; Williamson et al., 2014
	Lethrinus obsoletus	Mesopredator	Red Sea and E. Africa to Tonga and Samoa – S.W. Japan and Micronesia to Australia and New Caledonia	Seagrass beds and sand and rubble areas of coastal reefs, lagoons and outer slopes	0 – 30 m	Allen et al., 2003; Williamson et al., 2014
	Lethrinus ornatus	Mesopredator	Sri Lanka to Papua New Guinea – S.W. Japan to Great Barrier Reef	Seagrass beds and sand and rubble areas of coastal reefs, lagoons	0 – 30 m	Allen et al., 2003; Williamson et al., 2014
	Monotaxis grandoculis	Mesopredator	Red Sea and E. Africa to Micronesia, Hawaii and French Polynesia – S.W. Japan to Australia and New Caledonia	Coastal reefs lagoons and outer slopes	0 – 100 m	Allen et al., 2003; Williamson et al., 2014
**Lutjanidae**	*Lutjanus argentimaculatus*	Mesopredator	Red Sea and E. Africa to Samoa and Line Is. in eastern Central Pacific – S.W. Japan to S.E. Australia	Mangrove coasts to steep outer reefs	1 – 120 m	Allen et al., 2003; Williamson et al., 2014
	Lutjanus carponotatus	Mesopredator	India to Papua New Guinea and Solomon Is. – S. China to N. Australia and Great Barrier Reef	Turbid coastal reefs, lagoons and outer reef slopes	1 – 35 m	Allen et al., 2003; Williamson et al., 2014
	Lutjanus fulviflamma	Mesopredator	Red Sea and E. Africa to Samoa and Tonga – Taiwan to S.W. & S.E. Australia and Lord Howe I.	Estuaries, coastal reefs and outer slopes	3 – 35 m	Allen et al., 2003; Williamson et al., 2014
	Lutjanus fulvus	Mesopredator	Red Sea and E. Africa to Micronesia, Tuamotu and Marquesas and Rapa Is. in French Polynesia – S.W. Japan to S.E. Australia and Norfolk Is.	Coastal reefs, lagoons and outer reef slopes in 1 – 75 m	N/A	Allen et al., 2003; Williamson et al., 2014
	Lutjanus lemniscatus	Mesopredator	India and Sri Lanka to S. Papua New Guinea and N. Australia including Great Barrier Reef	Coastal reefs, lagoons and outer slopes	2 – 80 m	Allen et al., 2003; Williamson et al., 2014
	Lutjanus lutjanus	Mesopredator	Red Sea and E. Africa to Solomon Is. – S.W. Japan to Australia	Coastal reefs and outer slopes	10 – 90 m	Allen et al., 2003; Williamson et al., 2014

	Lutjanus monostigma	Mesopredator	Red Sea and E. Africa to Micronesia, Line Is. Tuamotu and Marquesas Is. in French Polynesia – S.W Japan to Australia and New Caledonia	Outer reef areas	5 – 60 m	Allen et al., 2003; Williamson et al., 2014
	Lutjanus quinquelineatus	Mesopredator	Arabian Gulf to Fiji – S.W. Japan to Australia	Coastal reefs, lagoons and outer reef slopes	2 – 40 m	Allen et al., 2003; Williamson et al., 2014
	Lutjanus russelli	Mesopredator	Red Sea and E. Africa to Fiji – S.W. Japan to Australia	Estuaries and coastal reefs	0 – 80 m	Allen et al., 2003; Williamson et al., 2014
	Lutjanus sebae	Mesopredator	Red Sea and E. Africa to Australia and Papua New Guinea, north to S. Japan	Deep, sandy bottoms	0 – 100m	Allen et al., 2003; Williamson et al., 2014
	Lutjanus vitta	Mesopredator	Seychelles and W. India to Marshall Is. in Micronesia and Gilbert Is. – S.W. Japan to Australia	Coastal and offshore reefs	10 – 72 m	Allen et al., 2003; Williamson et al., 2014
	Symphorus nematophorus	Mesopredator	S.W. Japan to N. Australia and New Caledonia	Coastal reefs	0 – 50 m	Allen et al., 2003; Williamson et al., 2014
**Mullidae**	*Parupeneus barberinus*	Benthic carnivore	E. Africa to Marquesas and Rapa Is. in French Polynesia – S. Japan to N. Australia an Vanuatu	Sand and rubble bottoms near reefs	0 – 100 m	Allen et al., 2003; Williamson et al., 2014
	Parupeneus ciliatus	Benthic carnivore	E. Africa to Line Is. in Marquesas and Rapa Is. in French Polynesia – S. Japan to Australia	Lagoons, seaward reefs and seagrass beds	0 – 40 m	Allen et al., 2003; Williamson et al., 2014
	Parupeneus indicus	Benthic carnivore	E. Africa and Arabian Sea to Samoa – S. Japan to Great Barrier Reef	Coastal reefs, lagoons and outer slopes	0 – 113 m	Allen et al., 2003; Williamson et al., 2014
**Muraenidae**	*Echidna nebulosa*	Mesopredator	Red Sea and E. Africa to Hawaii and Panama – S.W. Japan to Australia	Reef flats and rocky shorelines	1 – 18 m	Allen et al., 2003; Williamson et al., 2014
	Gymnothorax favagineus	Mesopredator	E. Africa and Oman to Australia and Papua New Guinea	Reef crevices of lagoon and outer reefs	1 – 50 m	Allen et al., 2003; Williamson et al., 2014
	Gymnothorax javanicus	Mesopredator	Red Sea and E. Africa to Hawaii and Pitcairn Is. in southeast Pacific, north to S.W. Japan	Reef holes of lagoon and outer reefs	1 – 46 m	Allen et al., 2003; Williamson et al., 2014
	Gymnothorax meleagris	Mesopredator	E. Africa to Hawaii and Galapagos Is. – S. Japan to Australia	Lagoon and outer reefs	1 – 36 m	Allen et al., 2003; Williamson et al., 2014
**Nemipteridae**	*Scolopsis bilineatus*	Mesopredator	Maldives to Fiji – S.W. Japan to Australia and New Caledonia	Sand and rubble fringe of reefs	0 – 25 m	Allen et al., 2003; Williamson et al., 2014
	Scolopsis margaritifer	Mesopredator	Sumatra in Indonesia to Palau and Vanuatu – Taiwan to N. Australia	Sand and rubble fringe of coastal reefs and lagoons	2 – 25 m	Allen et al., 2003; Williamson et al., 2014
	Scolopsis monogramma	Mesopredator	Sumatra in Indonesia to Papua New Guinea – S.W. Japan to E. Australia and New Caledonia	Sandy fringe of coastal reefs lagoons	2 – 50 m	Allen et al., 2003; Williamson et al., 2014
**Orectolobidae**	*Eucrossorhinus dasypogon*	Apex predator	N. Australia, Indonesia and S. New Guinea	Sheltered coastal and barrier reefs	1 – 15 m	Allen et al., 2003
	Orectolobus maculatus	Apex predator	S. & E. Australia	Coral and rocky reefs	0 – 110 m	Allen et al., 2003
	Orectolobus ornatus	Apex predator	Indonesia, New Guinea and Australia	Coral are rocky reefs	0 – 30 m	Allen et al., 2003
	Orectolobus wardi	Apex predator	Northern half of Australia	Coastal and lagoon reefs	0 – 5 m	Allen et al., 2003
**Pomacanthidae**	*Centropyge bicolor*	Soft coral/sponge feeder	Indonesia and Philippines, New Guinea to Samoa and Phoenix Is. – S.W. Japan to N.W. & E. Australia	Coral-rich and rubble areas of seaward reefs and lagoons	10 – 25 m	Allen et al., 2003
	Centropyge bispinosus	Soft coral/sponge feeder	E. Africa to Indonesia, Philippines, New Guinea, Micronesia and French Polynesia – S.W. Japan to E. Australia	Lagoons and Outer reef slopes	5 – 45 m	Allen et al., 2003
	Centropyge nox	Soft coral/sponge feeder	Indonesia, Philippines to Fiji and Vanuatu – S.W. Japan to N. Great Barrier Reef and New Caledonia	Coral-rich areas or rubble bottoms, sheltered outer reefs, lagoons and passes	10 – 70 m	Allen et al., 2003
	Centropyge tibicen	Soft coral/sponge feeder	Indonesia, Malaysian Peninsula, W. Pacific Rim, Philippines and New Guinea to New Caledonia – S.W. Japan to N. & E. Australia and Lord Howe I.	Coral and rubble areas of lagoons and seaward reefs	4 – 35 m	Allen et al., 2003
	Centropyge vrolikii	Soft coral/sponge feeder	Indonesia, Philippines and New Guinea to Marshall Is. and Vanuatu – S.W. Japan to E. Australia and Lord Howe I.	Sheltered coastal reefs and outer slopes	0 – 25 m	Allen et al., 2003
	Chaetodontoplus douboulayi	Soft coral/sponge feeder	N.W. & N.E. Australia, Aru Is. in Indonesia to S. New Guinea	Coastal reefs	5 – 20 m	Allen et al., 2003
	Chaetodontoplus meredithi	Soft coral/sponge feeder	E. Australia from Great Barrier Reef (rare) to Sydney area and Lord Howe I.	Flat bottoms with rocky coral patches	6 – 45 m	Allen et al., 2003
	Pomacanthus imperator	Soft coral/sponge feeder	Red Sea and E. Africa to Indonesia, Philippines, Hawaii and French Polynesia – S.W. Japan and N. & E. Australia	Coral reefs	6 – 60 m	Allen et al., 2003
	Pomacanthus semicirculatus	Soft coral/sponge feeder	E. Africa to Indonesia, Philippines, New Guinea and Fiji – S.W. Japan to N. W. & E. Australia	Sheltered inshore reefs	0 – 40 m	Allen et al., 2003
	Pomacanthus sexstriatus	Benthic carnivore	Indonesia, Philippines and New Guinea to New Caledonia – S.W. Japan to N.W. & E. Australia	Coastal, lagoon and outer reefs	3 – 60 m	Allen et al., 2003; Williamson et al., 2014
	Pomacanthus xanthometapon	Soft coral/sponge feeder	Maldives to Indonesia, Philippines, New Guinea and Vanuatu – S.W. Japan to Great Barrier Reef	Coral-rich areas	5 – 30 m	Allen et al., 2003
	Pygoplites diacanthus	Benthic carnivore	Red Sea and E. Africa to Indonesia, Philippines and Polynesia – S.W. Japan to N. Australia and New Caledonia	Lagoons and outer reefs	0 – 48 m	Allen et al., 2003; Williamson et al., 2014
**Pomacentridae**	*Abudefduf bengalensis*	Omnivore	Pakistan to Great Barrier Reef, N. to S.W. Japan	Costal and lagoon reefs	0 – 6 m	Allen et al., 2003; Williamson et al., 2014
	Abudefduf sexfasciatus	Omnivore	Red Sea and E. Africa to Rapa I. in S. French Polynesia	Coastal and offshore reefs	0 – 15 m	Allen et al., 2003; Williamson et al., 2014
	Abudefduf vaigiensis	Omnivore	Red Sea and E. Africa to Line Is. and French Polynesia – S.W. Japan to Great Barrier Reef	Shoreline reefs and outer slopes	0 – 12 m	Allen et al., 2003; Williamson et al., 2014
	Abudefduf whitleyi	Omnivore	Great Barrier Reef, Coral Sea and New Caledonia	Outer edge of reefs and surge gutters	0 – 5 m	Allen et al., 2003; Williamson et al., 2014
	Acanthochromis polyacanthus	Omnivore	Indonesia and Philippines to N. Australia and Solomon Is.	Shoreline, lagoon and outer reefs	0 – 65 m	Allen et al., 2003; Williamson et al., 2014

Amblyglyphidodon aureus	Omnivore	Andaman Sea to Marshall Is. in Micronesia and Fiji – S.W. Japan to N.W. Australia and New Caledonia	Steep outer reef slope	12 – 35 m	Allen et al., 2003; Williamson et al., 2014
Amblyglyphidodon curacao	Omnivore	Singapore to Vanuatu, Samoa and Marshall Is. – S.W. Japan to N.W. Australia and Great Barrier Reef	Lagoons and outer slopes	0 – 15 m	Allen et al., 2003; Williamson et al., 2014
Amblyglyphidodon leucogaster	Omnivore	N. Sumatra in Indonesia to Vanuatu – S.W. Japan to Great Barrier Reef	Lagoons and outer reefs	2 – 45 m	Allen et al., 2003; Williamson et al., 2014
Amphiprion akindynos	Omnivore	Great Barrier Reef, Coral Sea, New Caledonia and Loyalty Is.	Live with anemone species	3 – 25 m	Allen et al., 2003; Williamson et al., 2014
Chromis amboinensis	Planktivore	Cocos-Keeling Is. to Indonesia and Samoa and Mariana Is. in N. Micronesia	Lagoons and outer reefs	5 – 65 m	Allen et al., 2003; Williamson et al., 2014
Chromis atripectoralis	Planktivore	Mascarene and Seychelles Is. to Pitcairn Is. east of French Polynesia – S.W. Japan to Great Barrier Reef and Lord Howe I.	Lagoons and outer reefs	2 – 15 m	Allen et al., 2003; Williamson et al., 2014
Chromis atripes	Planktivore	Indonesia to Marshall Is. and Fiji – S.W. Japan to N.W. and N.E. Australia and New Caledonia	Steep outer reef slopes and deeper patch reefs	10 – 35 m	Allen et al., 2003; Williamson et al., 2014
Chromis nitida	Planktivore	Central and S. Great Barrier Reef, south to Sydney and Lord Howe I.	Lagoons and outer reefs in coral areas	5 – 25 m	Allen et al., 2003; Williamson et al., 2014
Chromis retrofasciatus	Planktivore	Indonesia and Philippines to Great Barrier Reef and Fiji	Bottom of lagoons an outer reefs	5 – 65 m	Allen et al., 2003; Williamson et al., 2014
Chromis ternatensis	Planktivore	E. Africa to Samoa – S.W. Japan to Great Barrier Reef and New Caledonia	Areas of *Acropora* corals	2 – 15 m	Allen et al., 2003; Williamson et al., 2014
Chromis weberi	Planktivore	Red Sea and E. Africa to Line Is. and Pitcairn Is. east of French Polynesia – S.W. Japan to Australia and New Caledonia	Coastal and outer reefs	3 – 25 m	Allen et al., 2003; Williamson et al., 2014
Chrysiptera rex	Omnivore	Spratly Is. in S. China Sea to N. Sulawesi in Indonesia including Togean and Banggai Is.	Reef tops and surge channels of fringing and offshore reefs	2 – 8 m	Allen et al., 2003; Williamson et al., 2014
Chrysiptera rolandi	Omnivore	Andaman Sea to Coral Sea, north to Philippines	Protected shoreline reefs, lagoons and outer slopes	2 – 35 m	Allen et al., 2003; Williamson et al., 2014
Chrysiptera talboti	Omnivore	Andaman Sea to Coral Sea and Fiji, north to Philippines	Shoreline reefs, lagoons and outer slopes	6 – 35 m	Allen et al., 2003; Williamson et al., 2014
Dascyllus aruanus	Omnivore	Red Sea and E. Africa to Line Is. and E. French Polynesia – S.W. Japan to Australia	Inshore and lagoon reefs	0 – 12 m	Allen et al., 2003; Williamson et al., 2014
Dischistodus melanotus	Territorial farmer	Indonesia to Great Barrier Reef and Coral Sea, north to S.W. Japan	Lagoon and inshore coral reefs	0 – 10 m	Allen et al., 2003; Williamson et al., 2014
Hemiglyphidodon plagiometapon	Territorial farmer	Andaman Sea, Indonesia, Philippines, New Guinea, Solomon Is. and Great Barrier Reef	Sheltered shoreline reefs and lagoons, often in areas of silting and turbid waters	0 – 20 m	Allen et al., 2003; Williamson et al., 2014
Neoglyphidodon melas	Territorial farmer	Red Sea and E. Africa to Vanuatu – S.W. Japan to N. Australia	Shoreline reefs, lagoons and outer slopes	0 – 12 m	Allen et al., 2003; Williamson et al., 2014
Neoglyphidodon nigroris	Territorial farmer	Andaman Sea to Great Barrier Reef, Coral Sea and Vanuatu, North to Palau and S.W. Japan	Passes and on outer reef slopes	2 – 23 m	Allen et al., 2003; Williamson et al., 2014
Plectroglyphidodon dickii	Territorial farmer	E. Africa to Line Is. and French Polynesia – S.W. Japan to Australia	Coral-rich areas of lagoons and outer reefs	0 – 12 m	Allen et al., 2003; Williamson et al., 2014
Plectroglyphidodon lacrymatus	Territorial farmer	E. Africa to Marshall Is. in Micronesia and French Polynesia – S.W. Japan to E. Australia	Lagoons and outer reefs	2 – 12 m	Allen et al., 2003; Williamson et al., 2014
Pomacentrus adelus	Territorial farmer	Andaman Sea to Great Barrier Reef and Coral Sea, north to Philippines	Inshore and outer reefs	0 – 8 m	Allen et al., 2003; Williamson et al., 2014
Pomacentrus amboinensis	Omnivore	Andaman Sea to Fiji and Marshall Is. in Micronesia – S.W. Japan to Australia	Sandy areas	2 – 40 m	Allen et al., 2003; Williamson et al., 2014
Pomacentrus australis	Omnivore	Great Barrier Reef to Sydney, Australia	Coral rock outcroppings in sand or rubble areas	5 – 35 m	Allen et al., 2003; Williamson et al., 2014
Pomacentrus bankanensis	Territorial farmer	Andaman Sea to Vanuatu and Fiji – S.W. Japan to Australia and Coral Sea	Lagoon and outer reefs	0 – 12 m	Allen et al., 2003; Williamson et al., 2014
Pomacentrus brachialis	Omnivore	Indonesia and Philippines to Great Barrier Reef, Coral Sea and Fiji, north to S.W. Japan	Passages and outer reef slopes	6 – 40 m	Allen et al., 2003; Williamson et al., 2014
Pomacentrus chrysurus	Territorial farmer	Maldives and Sri Lanka to Australia and New Caledonia, north to S.W. Japan	Sandy areas of shoreline reefs and lagoons	0 – 3 m	Allen et al., 2003; Williamson et al., 2014
Pomacentrus coelestis	Omnivore	Sri Lanka and Andaman Sea to Line Is. and French Polynesia, north to S.W. Japan	Rubble areas	0 – 12 m	Allen et al., 2003; Williamson et al., 2014
Pomacentrus lepidogenis	Planktivore	Andaman Sea to Philippines, Palau, Great Barrier Reef, Coral Sea, Fiji and Tonga	Shoreline, lagoon and outer reefs	0 – 12 m	Allen et al., 2003; Williamson et al., 2014
Pomacentrus moluccensis	Omnivore	Andaman Sea to Palau and Fiji – S.W. Japan to N.W. and E. Australia and Coral Sea	Shoreline, lagoon and outer reefs	0 – 14 m	Allen et al., 2003; Williamson et al., 2014
Pomacentrus nagasakiensis	Omnivore	Maldives to Palau and Vanuatu – S.W. Japan to N.W. Australia and New Caledonia	Sandy areas around rock outcroppings	5 – 30 m	Allen et al., 2003; Williamson et al., 2014
Pomacentrus vaiuli	Territorial farmer	Bali in Indonesia to Micronesia and Samoa – S.W. Japan to Australia	Lagoons and outer reef slopes	3 – 45 m	Allen et al., 2003; Williamson et al., 2014
Pomacentrus wardi	Territorial farmer	Great Barrier Reef and E. Australian coast south to Sydney area	Coastal and offshore reefs	0 – 20 m	Allen et al., 2003; Williamson et al., 2014

	Stegastes apicalis	Territorial farmer	Great Barrier Reef and E. Australian coast to Sydney area	Coastal reefs and inner-shelf of the Great Barrier Reef	0 – 5 m	Allen et al., 2003; Williamson et al., 2014
	Stegastes fasciolatus	Territorial farmer	E. Africa, Micronesia to Hawaii and Easter I. in eastern Pacific – S.W. Japan to and Australia and Lord Howe I.	Rock and coral reefs exposed to surge	0 – 5 m	Allen et al., 2003; Williamson et al., 2014
**Rhincodontidae**	*Rhincodon typus*	Planktivore	Circumtropical	Open clear waters or around reefs	0 – 40 m	Allen et al., 2003
**Rhinidae**	*Rhina ancylostoma*	Apex predator	E. Africa and Red Sea to Australia and New Guinea, north to Japan	Coastal reefs	3 – 90 m	Allen et al., 2003
	Rhynchobatus australiae	Apex predator	Andaman Sea to Philippines, south to Australia	Sandy areas in lagoons and around reefs	0 – 50 m	Allen et al., 2003
**Scaridae**	*Bolbometapon muricatum*	Excavator	Red Sea and E. Africa to Line Is. and Tuamotu Is. in French Polynesia – S.W. Japan to Australia	Coral lagoons and seaward reefs	0 – 40 m	Allen et al., 2003; Williamson et al., 2014
	Chlorurus bleekeri	Excavator	Indonesia to Vanuatu and Fiji – Philippines and Marshall Is. in Micronesia to E. Australia	Sheltered coastal reefs, lagoons and outer slopes	3 – 35 m	Allen et al., 2003; Williamson et al., 2014
	Chlorurus microrhinus	Excavator	Bali in Indonesia and Philippines to Line Is. and Pitcairn Is. east of French Polynesia – S.W. Japan to Australia	Sheltered reefs	0 – 50 m	Allen et al., 2003; Williamson et al., 2014
	Chlorurus sordidus	Excavator	Red Sea and E. Africa to Hawaii and Ducie Is. east of French Polynesia – S.W. Japan to Australia	Coral reefs and adjacent rubble	0 – 30 m	Allen et al., 2003; Williamson et al., 2014
	Hipposcarus longiceps	Excavator	Indonesia to Line Is. and Tuamotu Is. In French Polynesia – S.W. Japan to Australia	Lagoons and seaward reefs on sand bottoms near reefs	2 – 40 m	Allen et al., 2003; Williamson et al., 2014
	Scarus altipinnis	Scraper	S.W. Japan to Great Barrier Reef and Coral Sea, to Line Is. and Ducie I. east of French Polynesia	Shallow protected reefs and outer slopes	0 – 30 m	Allen et al., 2003; Williamson et al., 2014
	Scarus chamaeleon	Scraper	Indonesia and Philippines to Fiji – S.W. Japan to Australia	Coral-rich areas of lagoon and seaward reefs	0 – 35 m	Allen et al., 2003; Williamson et al., 2014
	Scarus dimidiatus	Scraper	Indonesia and Philippines to Samoa – S.W. Japan to Australia and Vanuatu	Lagoon and seaward reefs	0 – 25 m	Allen et al., 2003; Williamson et al., 2014
	Scarus flavipectoralis	Scraper	Andaman Sea to Solomon Is. – Marshall Is. In Micronesia to Great Barrier Reef and New Caledonia	Coastal, lagoon and outer reefs	8 – 40 m	Allen et al., 2003; Williamson et al., 2014
	Scarus frenatus	Scraper	Red Sea and E. Africa to Line Is. And Ducie Is. east of French Polynesia – S.W. Japan to Australia	Seaward slopes and reef crests	0 – 25 m	Allen et al., 2003; Williamson et al., 2014
	Scarus ghobban	Scraper	Red Sea and E. Africa to French Polynesia, Galapagos, Gulf of California and Panama – S. Japan to Australia	Sheltered silty inshore reefs and areas of sand and rubble	2 – 30 m	Allen et al., 2003; Williamson et al., 2014
	Scarus globiceps	Scraper	E. Africa to Line Is. And French Polynesia – S.W. Japan to Australia	Reef flats, lagoons and seaward reefs	0 – 30 m	Allen et al., 2003; Williamson et al., 2014
	Scarus niger	Scraper	Red Sea and E. Africa to Society Is. in French Polynesia – S.W. Japan to Australia	Coral-rich areas	0 – 20 m	Allen et al., 2003; Williamson et al., 2014
	Scarus psittacus	Scraper	Red Sea to Hawaii and French Polynesia – S. Japan to S.E. Australia and Lord Howe I.	Reef flats, lagoons and seaward slopes	2 – 25 m	Allen et al., 2003; Williamson et al., 2014
	Scarus rivulatus	Scraper	Andaman Sea to Australia and New Caledonia, north to S.W. Japan	Silty coastal reefs, lagoons and seaward reefs	0 – 20 m	Allen et al., 2003; Williamson et al., 2014
	Scarus rubroviolaceus	Scraper	E. Africa to Hawaii, Tuamotu Is. in French Polynesia, Galapagos and Panama – S.W. Japan to Australia	Outer reef slopes	0 – 30 m	Allen et al., 2003; Williamson et al., 2014
	Scarus schlegeli	Scraper	E. Indonesia to French Polynesia – S.W. Japan to Australia	Coral-rich areas of coastal, lagoon and outer reefs	0 – 50 m	Allen et al., 2003; Williamson et al., 2014
	Scarus spinus	Scraper	Indonesia and Philippines to Samoa – S.W. Japan to Great Barrier Reef	Outer reefs	2 – 25 m	Allen et al., 2003; Williamson et al., 2014
	Scarus tricolor	Scraper	E. Africa to Palau, Papua New Guinea, Nauru and Line Is. in eastern Central Pacific	Outer slopes	10 – 40 m	Allen et al., 2003; Williamson et al., 2014
**Serranidae**	*Aethaloperca rogga*	Mesopredator	Red Sea and E. Africa to Fiji – S.W. Japan to Australia	Inside or near caves or under ledges in coral rich areas of seaward reefs	3 – 50 m	Allen et al., 2003; Williamson et al., 2014
	Anyperodon leucogrammicus	Mesopredator	Red Sea and E. Africa to Marshall Is. in Micronesia and Samoa – S.W. Japan to Australia	Sheltered coastal and outer reefs	5 – 80 m	Allen et al., 2003; Williamson et al., 2014
	Cephalopholis boenak	Mesopredator	E. Africa to Australia and New Caledonia, north to S.W. Japan	Dead silty reefs in sheltered waters and coastal reefs	4 – 30 m	Allen et al., 2003; Williamson et al., 2014
	Cephalopholis cyanostigma	Mesopredator	E. Malaysian Peninsula, Philippines and Papua New Guinea to Solomon Is. and Great Barrier Reef	Coastal lagoon and seaward reefs	1 – 50 m	Allen et al., 2003; Williamson et al., 2014
	Cephalopholis microprion	Mesopredator	Malaysian Peninsula and Philippines to Great Barrier Reef and Fiji	Dead silty reefs in sheltered coastal waters	2 – 20 m	Allen et al., 2003; Williamson et al., 2014
	Cromileptes altivelis	Mesopredator	E. Malaysian Peninsula to Australia and New Caledonia, north to S.W. Japan	Dead, silty reefs	1 – 30 m	Allen et al., 2003; Williamson et al., 2014
	Diploprion bifasciatus	Mesopredator	India and Maldives to Papua New Guinea – S.W. Japan to Australia and Vanuatu	Coastal reefs	1 – 18 m	Allen et al., 2003; Williamson et al., 2014
	Epinephelus caerulopunctatus	Mesopredator	E. Africa and Arabian Gulf to Fiji – S.W. Japan to E. Australia	Near caves of coastal, lagoon and seaward reefs	4 – 65 m	Allen et al., 2003; Williamson et al., 2014
	Epinephelus fasciatus	Mesopredator	Red Sea and E. Africa to Pitcairn Is. east of French Polynesia	Coastal, lagoon and seaward reefs	3 – 160 m	Allen et al., 2003; Williamson et al., 2014
	Epinephelus fuscoguttatus	Mesopredator	Red Sea E. Africa to Samoa – S.W. Japan to Australia	Coastal, lagoon and outer reef slopes	1 – 60 m	Allen et al., 2003; Williamson et al., 2014

	Epinephelus lanceolatus	Mesopredator	S. Red Sea and E. Africa to Pitcairn Is. east of French Polynesia – S.W. Japan to Australia	Coastal reefs, lagoons and outer slopes	3 – 100 m	Allen et al., 2003; Williamson et al., 2014
	Epinephelus merra	Mesopredator	E. Africa to Tuamotu Is. in French Polynesia – S.W. Japan to Australia	Coastal, lagoon and sheltered outer reefs	1 – 50 m	Allen et al., 2003; Williamson et al., 2014
	Epinephelus ongus	Mesopredator	E. Africa to Fiji – S.W. Japan to Australia	Near caves and ledges of coastal and lagoon reefs	5 – 25 m	Allen et al., 2003; Williamson et al., 2014
	Epinephelus quoyanus	Mesopredator	Andaman Sea to Papua New Guinea – S.W. Japan to Australia	Silty coastal reefs	1 – 50 m	Allen et al., 2003; Williamson et al., 2014
	Plectropomus laevis	Mesopredator	E. Africa to Tuamoutus Is. in French Polynesia – S.W. Japan to Great Barrier Reef	Lagoons and seaward reefs	4 – 90 m	Allen et al., 2003; Williamson et al., 2014
	Plectropomus leopardus	Mesopredator	South China Sea and S.W. Japan to Australia and Fiji	Coastal and lagoon reefs	3 – 100 m	Allen et al., 2003; Williamson et al., 2014
	Plectropomus maculatus	Mesopredator	Malaysian Peninsula and Philippines to Papua New Guinea and Australia	Silty coastal reefs	5 – 50 m	Allen et al., 2003; Williamson et al., 2014
**Siganidae**	*Siganus argenteus*	Algal cropper	Red Sea and E. Africa to French Polynesia – S.W. Japan to Great Barrier Reef and New Caledonia	Juveniles inshore, adults prefer outer reefs slopes	0 – 40 m	Allen et al., 2003; Williamson et al., 2014
	Siganus corallinus	Algal cropper	Seychelles to New Caledonia – S.W. Japan to S. Great Barrier Reef	Coral-rich areas	0 – 18 m	Allen et al., 2003; Williamson et al., 2014
	Siganus doliatus	Algal cropper	Sulawesi in Indonesia to Tonga – Palau to S. Great Barrier Reef and New Caledonia	Inshore and outer reefs	2 – 15 m	Allen et al., 2003; Williamson et al., 2014
	Siganus fuscescens	Algal cropper	Andaman Sea to Vanuatu and E. Micronesia – S.W. Japan to W. & E. Australia and New Caledonia	Coastal reefs, inner reefs of deep lagoons and sea grass beds	0 – 4 m	Allen et al., 2003; Williamson et al., 2014
	Siganus javus	Algal cropper	Arabian Gulf to Vanuatu – S. Japan and Philippines to N.E. Australia	Coastal reefs, occasionally brackish water mangroves	0 – 15 m	Allen et al., 2003; Williamson et al., 2014
	Siganus lineatus	Algal cropper	Maldives and Sri Lanka to Vanuatu – Borin Is. in S. Japan to N.W. Australia, S. Great Barrier Reef and New Caledonia	Lagoons, coastal reefs and mangroves	0 – 25 m	Allen et al., 2003; Williamson et al., 2014
	Siganus puellus	Algal cropper	Cocos-Keeling Is. to S. Micronesia and Gilbert Is. – S. China Sea and S.W. Japan to S. Great Barrier Reef, New Caledonia and Vanuatu	Rich-coral areas	3 – 12 m	Allen et al., 2003; Williamson et al., 2014
	Siganus punctatus	Algal cropper	Cocos-Keeling Is. to Sumatra in Indonesia, Micronesia and Samoa – S.W. Japan to W. & E. Australia and New Caledonia	Coral areas	1 – 40 m	Allen et al., 2003; Williamson et al., 2014
	Siganus spinus	Algal cropper	India to Society Is. in French Polynesia – S.W. Japan to S. Great Barrier Reef	Coastal reef flats and outer reefs	0 – 6 m	Allen et al., 2003; Williamson et al., 2014
	Siganus vulpinus	Algal cropper	Sumatra in Indonesia to Gilbert Is. – Taiwan to Great Barrier Reef and New Caledonia	Coral-rich areas of lagoons and outer reefs	0 – 30 m	Allen et al., 2003; Williamson et al., 2014
**Stegostomatidae**	*Stegostoma fasciatum*	Apex predator	Red Sea and E. Africa to Australia and New Caledonia, north to S. Japan	Coastal and offshore reefs	1 – 70 m	Allen et al., 2003
**Sphymidae**	*Sphyma lewini*	Apex predator	Tropical and warm temperate seas	Open water of seaward reefs, around seamounts and islands	1 – 275 m	Allen et al., 2003
	Sphyma mokarran	Apex predator	Tropical and warm temperate seas	Oceanic, rarely on reefs	3 – 75 m	Allen et al., 2003
**Zanclidae**	*Zanclus cornutus*	Soft coral/sponge feeder	E. Africa to Micronesia, Hawaii, Polynesia, Mexico and Galapagos – S.W. Japan to Great Barrier Reef	Lagoons and outer reefs	0 – 180 m	Allen et al., 2003; Williamson et al., 2014
