# Supplementary material for: Variability in the functional composition of coral reef fish communities on submerged and emergent reefs in the central Great Barrier Reef, Australia
Source: PLoS One. 2019 May 17;14(5):e0216785. doi: 10.1371/journal.pone.0216785 (PMC6524821; doi:10.1371/journal.pone.0216785)
Supplement: S2 Table — (PDF) [file pone.0216785.s002.pdf]

Reef Type	Shelf Position	Site	Depth (m)	# of Transects
Submerged Shoals	Mid-shelf	Isabella Shoal	10	4
			20	4
			30	3
		Lyrad Shoal	10	2
			20	4
			30	4
		Oropesa Shoal	10	3
			20	3
			30	1
		Stevens Shoal	10	4
			20	4
			30	4
	Outer-shelf	Done Shoal	10	4
			20	4
			30	2
		Jenny Louise Shoal	10	3
			20	4
			30	4
		Onyx Shoal	10	4
			20	4
			30	4
		Outer Shoal	10	3
			20	4
			30	4
Emergent Reefs		Hastings Reef	6	2
			10	2
			20	2
		Michaelmas Reef	6	2
			10	2
			20	2
